# Supplementary material for: Wnt signaling from Gli1-expressing apical stem/progenitor cells is essential for the coordination of tooth root development
Source: Stem Cell Reports. 2023 Mar 16;18(4):1015–29. doi: 10.1016/j.stemcr.2023.02.004 (PMC10147554; doi:10.1016/j.stemcr.2023.02.004)
Supplement: Document S1. Supplemental experimental methods and Figures S1–S7 [file mmc1.pdf]

Stem Cell Reports, Volume *18*

Supplemental Information

Wnt signaling from *Gli1*-expressing apical stem/progenitor cells is essential for the coordination of tooth root development

Rupali Lav, Jan Krivanek, Neal Anthwal, and Abigail S. Tucker

Supplemental Figures:

Figure S1:

***Axin2* lineage cells populate most mesenchymal cells of the incisor pulp and periodontium but not the epithelium**

(A) Schematic outlining the time course of tamoxifen (Tmx) administered to postnatal *Axin2*creERT2;R26mTmG mice for lineage tracing apical Wnt responding cells. (B) and magnified labial (B') and lingual (B'') cervical loops show sparce *Axin2* expressing cells (GFP) (24hrs post Tmx) found in the proximal incisor at P13. (C) and magnified labial (C') and lingual (C'') cervical loops show abundant *Axin2* lineage cells (GFP) populating the pulp and surrounding periodontium (PDL) but not the epithelial loops. n ≥ 3 tissue sections were examined from n= 5 mice. Scale bar in B,B' = 100μm, same scale in B' and C-C".

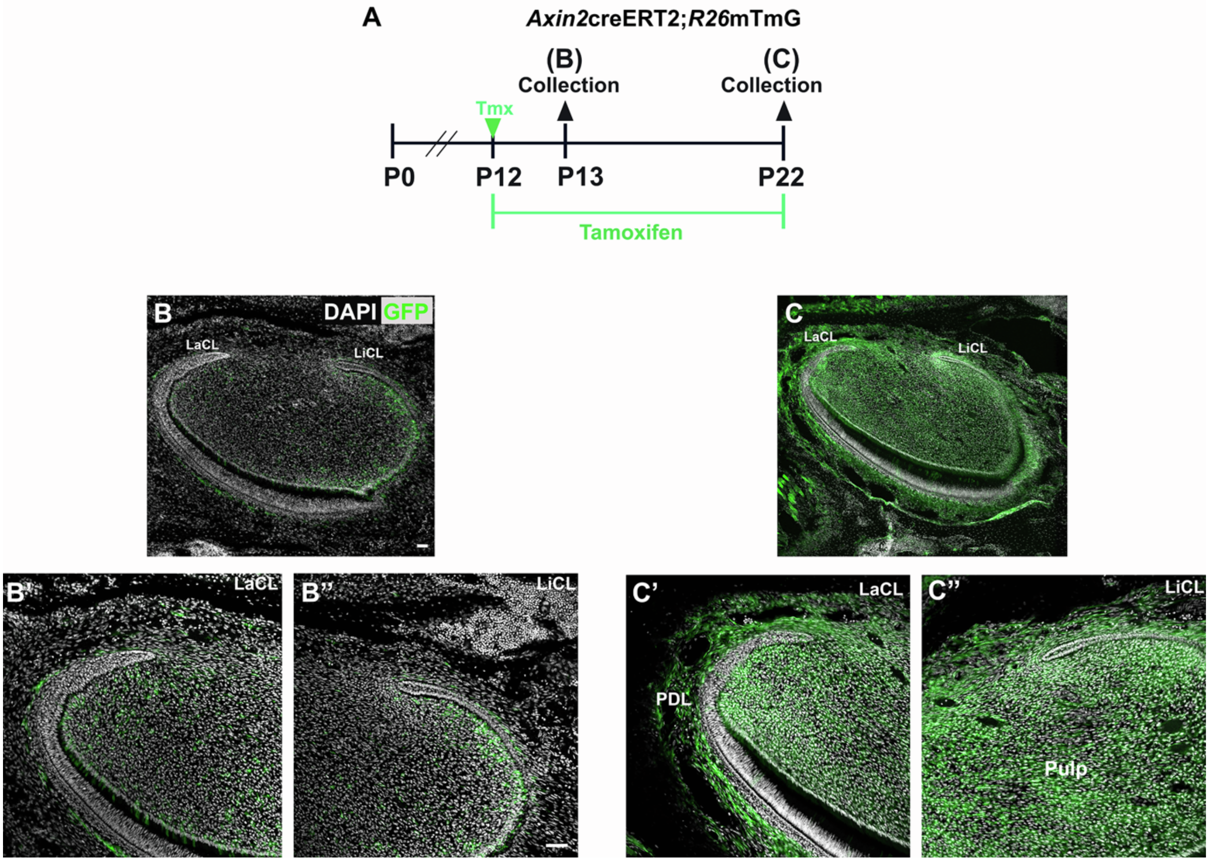

Figure S2:

Apical *Gli1*+ stem progenitor cells form all root lineage cells

(A) *Gli1*creERT2;R26Tomato reporter mice were used to labels stem/progenitor cells in the developing molar root. *Gli1* (detected through red fluorescent protein (RFP) expression- A') labels stem progenitor cells at the apex forming a proliferative (A''-Proliferative Cell Nuclear Antigen expression (PCNA)) progenitor cell population. Lineage traced *Gli1* progeny (RFP) (B and B'-magnified) form cells of all lineages in the root and surrounding periodontium (P-Pulp, Od-Odontoblasts, PDL-Periodontal Ligament, D-Dentin). n ≥ 3 tissue sections were examined from n= 3 mice. Scale bar in A", B and B'= 100μm, same scale in A-A'.

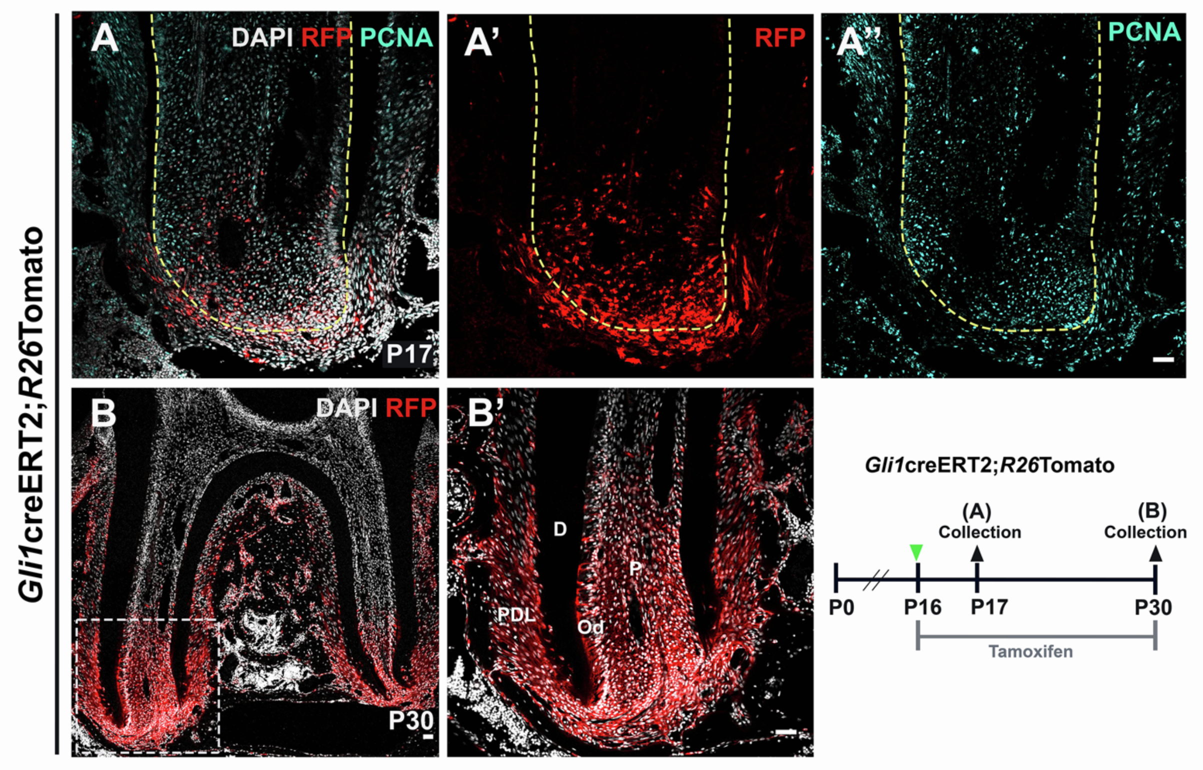

Figure S3:

Calvarial and molar defect in *Gli1*creERT2;*Wls*$^{fl/fl}$ mutant mice.

(A-C) RNAscope showing *Axin2* expression after loss of *Wntless* in the *Gli1* expressing population. (A) Apical region of molar (M1) P18 after tamoxifen at P13. (B) High power view of the apical region (box B in A) shows loss of *Axin2* expression (yellow dots). (C) High power view of periodontium (box C in A) shows high levels of *Axin2* expression (yellow dots). (D-I) microCT analysis of *Gli1creERT2;Wls*$^{fl/fl}$ mutant mice. Coronal (D,E) and sagittal (F-I). 3D microCT whole head reconstructions show that compared to littermate controls (D,F,H), *Gli1*creERT2;*Wls*$^{fl/fl}$ mutant mice exhibit rarefied bone especially adjacent to cranial sutures (E) and unerupted third molars (G), and truncated incisors (I). Asterix indicates defect in the lingual cervical loop. n= 5 mice. Scale bar in A-C = 50μm.

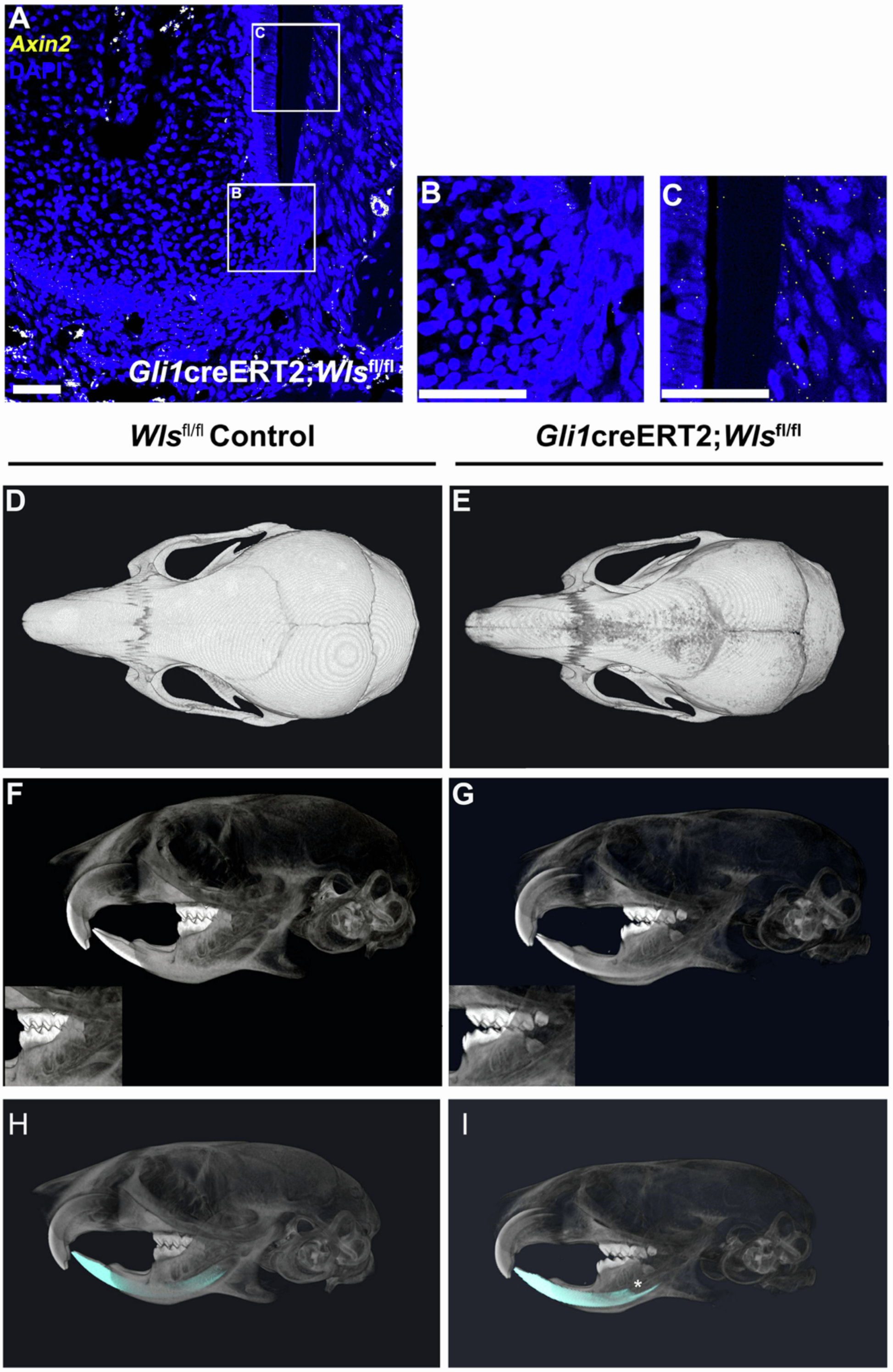

Figure S4:

Tooth root defects predate any evidence of bone defects at P18.

TRAP assay. Osteoclasts in red. (A,C,E,G) Wildtype littermate at P18, M1. (B,D,F,H) *Gli1creERT2;Wls$^{fl/fl}$* mutant mice at P18 after tamoxifen induction a P13, M1. Osteoclast activity appears normal at P18 after tamoxifen induction at P13. Scale bar in images = 50μm.

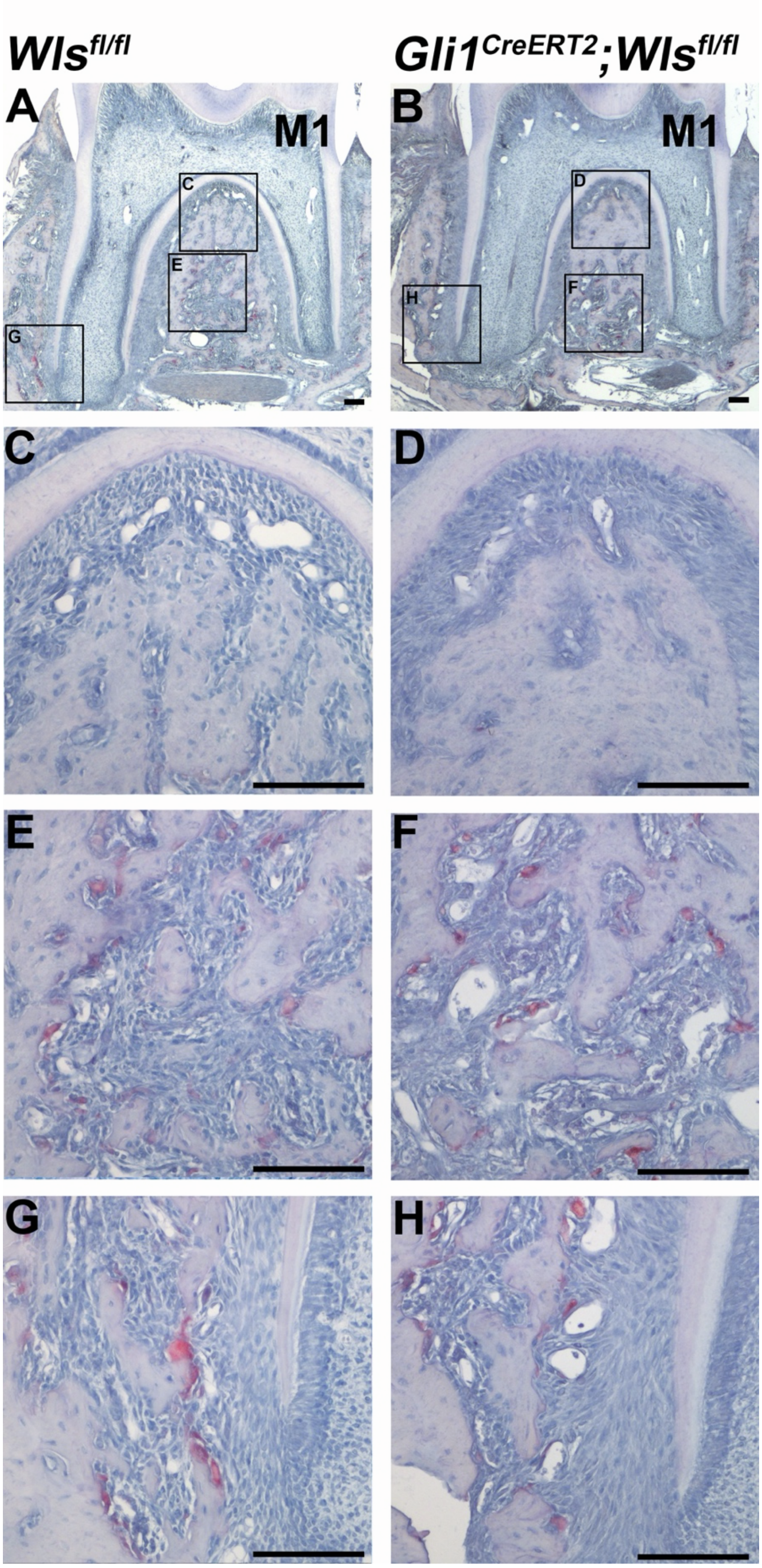

Figure S5:

Loss of Wnt ligands produced by *Gli1*⁺ cells resulted in reduced cell proliferation

(A,B) P20. (C,D) P18. (A,A'- magnified) BrdU (5-Bromo-2'deoxyuridine) uptake (pink) by numerous proliferating apical root forming cells in non-Cre littermate controls (white arrowheads). (B, B'-magnified) Selective absence of cell proliferation in the apical root forming cells in mutants with *Wls* deletion in *Gli1*$^{+}$ cells (asterix). Unaffected proliferating cells are observed in the surrounding periodontium and under the HERS (Hertwig's Epithelial Root Sheath) (yellow arrowheads). (C, C') Proliferating cells (pink) in the control HERS (yellow arrowheads). Epithelial cells highlighted by pan-cytokeratin staining (green). (D,D') Lack of proliferating cells in the HERS in the mutant.

n ≥ 3 tissue sections were examined from n≥ 3 mice each from mutant and control samples. Scale bar in A, A' and C, C'= 100µm, same scale in B, B' and D,D'.

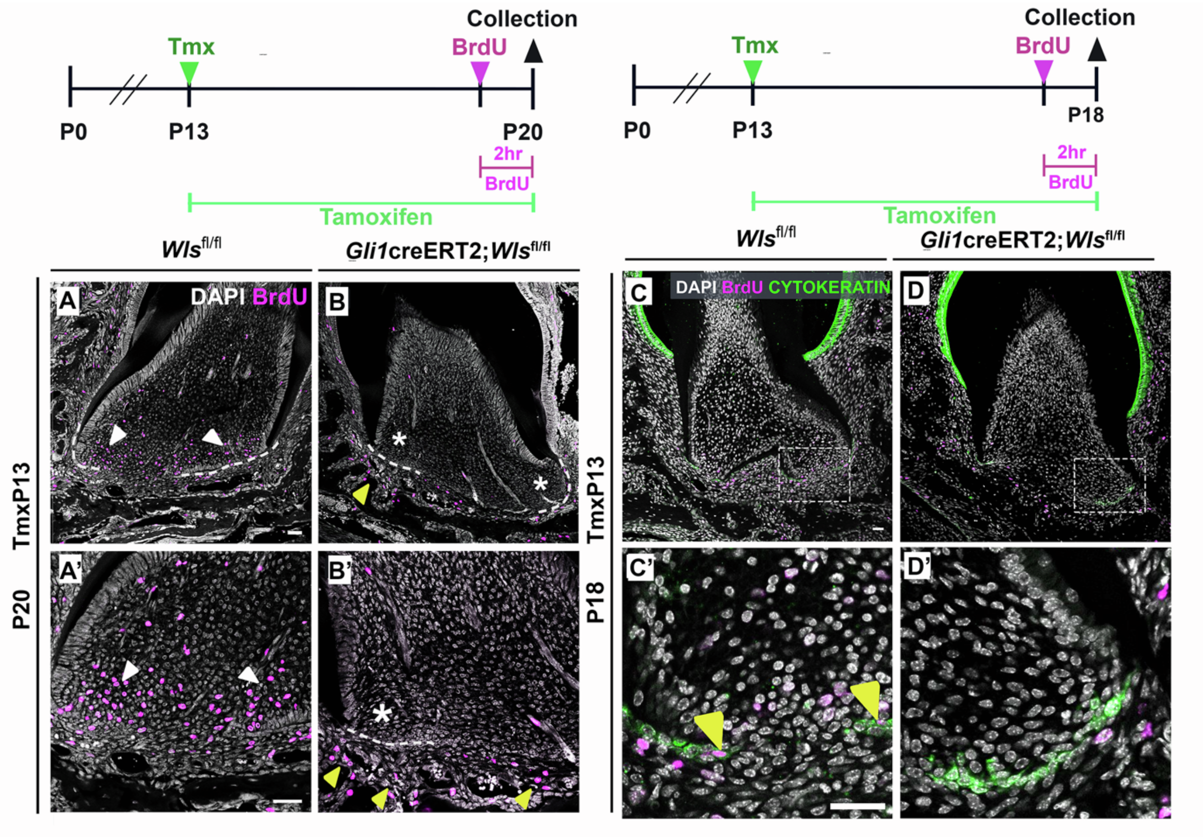

Figure S6:

Loss of Wnt ligands produced by *Gli1*⁺ cells resulted in loss of LAMININ expression and downregulation of Sox9 in the mutant HERS

LAMININ expression (pink) seen in the apical epithelium and mesenchyme of control developing roots (A, B-magnified) was lost in the mesenchyme of mutant molars (C,D-magnified). Low expression was seen in the apical HERS of mutant molars (D,D'). n ≥ 3 tissue sections were examined from n≥ 3 mice each from mutant and control samples

(E,G) Intact apical HERS cells of developing control molars labelled by E-CADHERIN (E-CAD) (green), with positive SOX9 cells (pink). Yellow arrowheads indicate SOX9 positive epithelial cells. (F,H) Mutant HERS lose expression of SOX9 (pink) and E-CADHERIN (green) (blue arrowhead). n ≥ 3 tissue sections were examined from n≥ 3 mice each from mutant and control samples. Scale bar in C,D,D',E,G= 100µm, same scale in A,B,B',F,H.

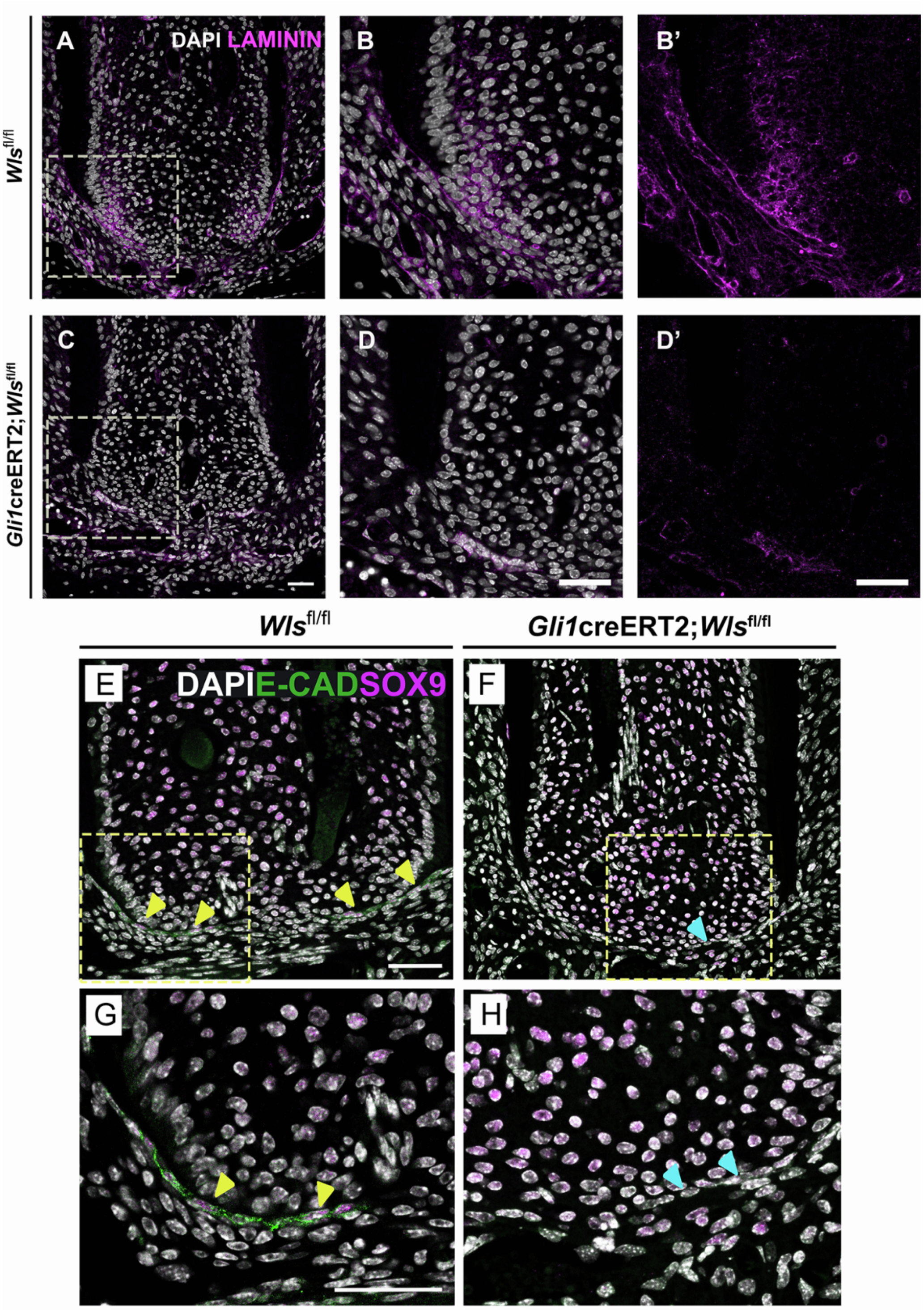

Figure S7:

***Sox9* deletion in *Gli1*⁺ stem cells effects developing molars but not homeostatic incisors**

(A) Deletion of *Sox9* in *Gli1* expressing cells results in overall growth stunting accompanied by thinning of skin and hair loss. (B,C) Loss of SOX9 protein in *Gli1*creERT2;*Sox9*$^{fl/fl}$ mutant (C) compared to control littermates (B). As compared to non-Cre littermate controls (D,F) molar (haematoxylin and eosin stained histology sections) roots are severely affected in mutant teeth (E,G). Homeostatic incisors (microCT 3D reconstructions) however show no evident morphological difference between control (H,J) and mutant (I,K) samples. n=6 mice each with mutant (*Gli1*creERT2;*Sox9*$^{fl/fl}$) and control (non-Cre littermates). Scale bar in B = 50μm, same scale in C. Scale bar in D,F = 100μm, same scale in E,G.

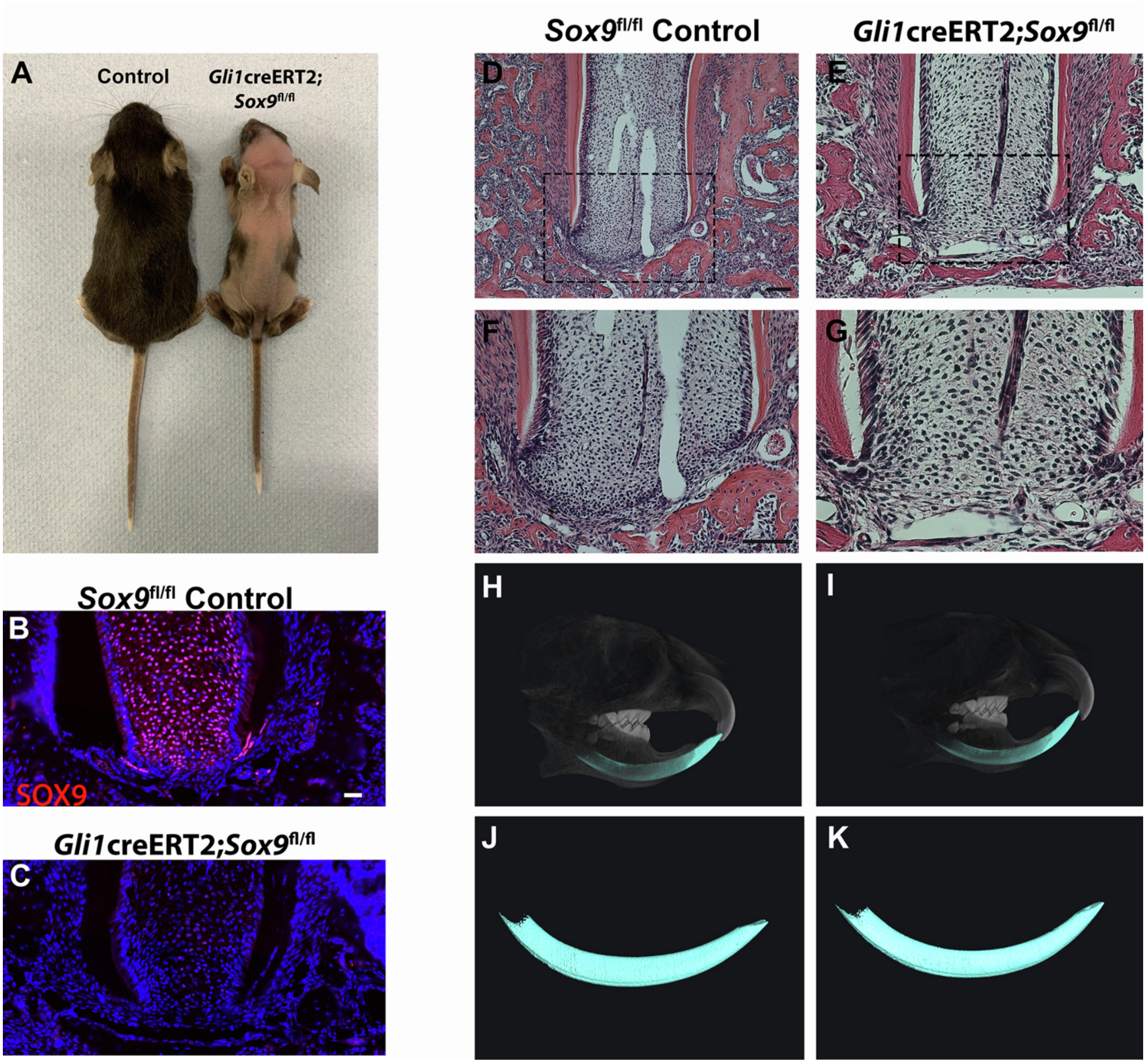

Supplemental Experimental methods:

Murine models:

All Cre mice were heterozygous for Cre, and loss of function experiments were performed in homozygous floxed models placed under temporally controlled Cre drivers. Gain of function experiments for constitutive overactivation of Wnt signaling were performed on heterozygous (*Ctnnb1*$^{lox(ex3)/+}$) mice.
For all inducible mouse lines, intraperitoneal injections of tamoxifen (Sigma T5648) dissolved in corn oil at 20 mg/ml with 10% ethanol were administered at indicated time points at a dose of 0.15 mg/g body weight. Additional BrdU (5-Bromo-2'deoxyuridine) (20mg/kg in normal saline) intraperitoneal injections were administered to *Gli1*creErt2;*Wls*$^{fl/fl}$ mice 2hrs prior to harvesting.

For all experiments, both female and male mice were non-preferentially used. Jaws were harvested at indicated time points post-induction and processed for further analysis.

Tissue processing and histological staining:

Mandibles with teeth were dissected and fixed overnight at 4°C in 4% paraformaldehyde (PFA), decalcified in 19% ethylenediaminetetraacetic acid (EDTA, pH 7.4), then dehydrated through an ascending series of graded ethanol, cleared with xylene, before wax infiltration with paraffin wax at 60°C. Paraffin wax-embedded samples were microtome sectioned at 8 μm thickness, then mounted on TruBond TM 380 adhesive slides. For histological examination, the slides were then stained with Hematoxylin and Eosin and/or picrosirius red trichrome stain using standard techniques. Stained sections were photographed using a Nikon eclipse 80i microscope.

X-gal staining and detection of β-galactosidase activity:

Postnatal day 16 (P16) murine *Axin2*LacZ mandibles were fixed in 0.2% glutaraldehyde overnight at 4°C, decalcified in 19% ethylenediaminetetraacetic acid (EDTA, pH 7.4) put through ascending graded sucrose series, embedded in OCT compound (Tissue-Tek) and sectioned with a cryostat at 10μm. For β-galactosidase (β-gal) activity detection, cryosections were washed in PBS, permeabilized in detergent rinse (1% Sodium Deoxycholate, 1M $MgCl_2$, 1% NP-40 in PBS, on ice for 10 min) and stained with X-gal staining solution [1M $MgCl_2$, 1 % sodium deoxycholate, 1% NP-40, 10 mM potassium ferricyanide, 10 mM potassium ferrocyanide, 1M Tris-HCL (pH 7.5) and 50 mg/ml X-gal (dissolved in Dimethyl formamide) in PBS] overnight at 37°C in a dark humidified chamber, followed by post-staining fixation in 4% PFA for 10 min at room temperature and counterstained with alcoholic Eosin histochemical stain. Stained sections were then flash dehydrated in ethanol, mounted in DPX and photographed using Nikon eclipse 80i microscope.

Immunofluorescence antibodies:

The following primary antibodies were used in this manuscript: 1/200 rabbit anti activated β-CATENIN (Cell Signaling, D13A1), 1/300 rabbit anti GFP (Invitrogen, A11122), 1/300 chicken anti GFP (Abcam ab13970), 1/300 rabbit anti PCNA (Abcam ab19166), 1/200 rat anti RFP (Chromotek, 5F8), 1/200 chicken anti GFP (Abcam, ab13970), 1/200 rat anti BrdU (Abcam, ab6326),1/200 rabbit anti pSMAD 1/5/8(Cell Signaling, D5B10), 1/300 rabbit anti CYOKERATIN (DAKO, Z0622), 1/200 mouse anti E-CADHERIN (Abcam, ab76055), 1/200 rabbit anti-LAMININ (Sigma Aldrich L9393), 1/200 rabbit anti SOX9 (Merck Millipore, AB5535), 1/200 goat anti-P-CADHERIN (R&D Systems, AF761;). Following TBS washes, sections were incubated with secondary antibody for 2 hours at room temperature. The following secondary antibodies were used at 1/300 concentration: Alexa568 conjugated Donkey anti-Rabbit (Invitrogen, A10042), Alexa 488 conjugated Donkey anti-Rabbit (Invitrogen, A21206), Alexa568 conjugated Donkey anti-Mouse (Invitrogen, A10037), Alexa594 conjugated Donkey anti-Rat (Invitrogen, A21209), Alexa488 conjugated Goat anti-Chicken (Invitrogen, A11039).
