# Supplementary material for: Wnt signaling from Gli1-expressing apical stem/progenitor cells is essential for the coordination of tooth root development
Source: Stem Cell Reports. 2023 Mar 16;18(4):1015–29. doi: 10.1016/j.stemcr.2023.02.004 (PMC10147554; doi:10.1016/j.stemcr.2023.02.004)
Supplement: Document S2. Article plus supplemental information [file mmc2.pdf]

Stem Cell Reports
Article
OPEN ACCESS

Wnt signaling from *Gli1*-expressing apical stem/progenitor cells is essential for the coordination of tooth root development

Rupali Lav,[1] Jan Krivanek,[2] Neal Anthwal,[1] and Abigail S. Tucker[1,*]
[1]Centre for Craniofacial and Regenerative Biology, King's College London, London, UK
[2]Department of Histology and Embryology, Faculty of Medicine, Masaryk University, Brno, Czech Republic
*Correspondence: abigail.tucker@kcl.ac.uk
https://doi.org/10.1016/j.stemcr.2023.02.004

SUMMARY

Stem cell regulation plays a crucial role during development and homeostasis. Here, an essential source of Wnts from *Gli1*$^+$ stem/progenitor cells was identified in the murine molar. Loss of Wnt production in *Gli1*$^+$ apical stem/progenitor cells led to loss of *Axin2* at the root apex, mis-regulation of SOX9, loss of BMP and Hh signaling, and truncation of root development. In the absence of Wnt signals, the root epithelium lost its integrity and epithelial identity. This phenotype could be partially mimicked by loss of *Sox9* in the *Gli1* population. Stabilization of Wnt signaling in the apical papilla led to rapid unordered differentiation of hard tissues and fragmentation of the epithelial root sheath. Wnt signaling from *Gli1*$^+$ stem/progenitor cells, therefore, orchestrates root development, coordinating mesenchymal and epithelial interactions via SOX9 to regulate stem/progenitor cell expansion and differentiation. Our results demonstrate that disparate stem/progenitor cell populations are unified in their fundamental signaling interactions.

INTRODUCTION

Wnt signaling is essential for stem cell self-renewal, proliferation, and differentiation (Clevers et al., 2014). This has been demonstrated in diverse tissues and organs such as bone (Liu et al., 2009), kidney (Barker et al., 2012), gut (Farin et al., 2016), and mammary gland (Plaks et al., 2013). In the gut, Wnts are key to maintaining the stem/progenitor cells at the base of the crypts. Wnt responding cells form all epithelial cell lineages found in the gut (Valenta et al., 2016). More recently, *Gli1*-expressing mesenchymal stem cells have been shown to secrete Wnt ligands crucial for the maintenance of the epithelial stem cell niche in the colon (Degirmenci et al., 2018). *Gli1* is a stem cell marker in many tissues including bone (Shi et al., 2017), kidney (Kramann et al., 2016), and gut (Valenta et al., 2016). Interestingly, Wnts and *Gli1* play important roles in tooth development. Wnts play diverse roles in specification and differentiation of odontogenic cells in early stages of dental organogenesis (Sarkar and Sharpe, 1999; Jussila and Thesleff, 2012). At later stages, Wnts are essential for root development. Both canonical (Lohi et al., 2009; Kim et al., 2013; Zhang et al., 2013; Bae et al., 2015) and non-canonical pathways (Ma et al., 2020) have been shown to be active during root development. In particular, Wnts are essential for the differentiation of odontoblasts forming root dentin (Kim et al., 2013). *Gli1* is an essential stem cell marker in the developing molar and can give rise to all lineages forming the postnatal molar root (Li et al., 2017).

Like many organ systems, teeth develop through a series of carefully orchestrated epithelial-mesenchymal interactions. Mice present two distinct dental developmental models: continuously growing non-rooted incisors and rooted molars. The mouse proximal incisors house a persistent stem cell niche that supports continuous odontogenesis, thus providing a good model system to study developmental mechanisms during homeostasis. The mouse molars, on the other hand, develop roots during early postnatal life with the organization of a transient stem/progenitor cell cluster at the base of the crown known as SCAPs (stem cells from the apical papilla). SCAPs are lost at the end of the first postnatal month, by which time the tooth has erupted into the oral cavity and root development is complete (Figure 1A). The apical papilla is enclosed on both sides by epithelial cells that create a structure called Hertwig's epithelial root sheath (HERS). HERS is bilayered and comprised of closely packed epithelial cells that are formed by apical proliferation in the cervical loop of the developing tooth germ (Zeichner-David et al., 2003; Huang et al., 2009a). HERS plays a crucial role in guiding root development and forms a physical boundary between the root-forming cells and the periodontium (Huang et al., 2009a; Li et al., 2017; Fons Romero et al., 2017). As roots near completion, most epithelial cells undergo apoptosis or epithelial-to-mesenchymal transition to form cementoblasts (Huang et al., 2009a). A small subset of these epithelial cells have been shown to consolidate and persist as epithelial cell rests of Malassez (ERMs), which may function as local stem cell niches within the periodontal ligament (Oka et al., 2012).

Given the relationship between Wnts and *Gli1* in the gut, the relationship between the *Gli1*-expressing cells and Wnt signaling was investigated during root development to explore their role in maintaining the molar root stem cell niche. As Wnts are important in gut and tooth

This is an open access article under the CC BY license (http://creativecommons.org/licenses/by/4.0/).

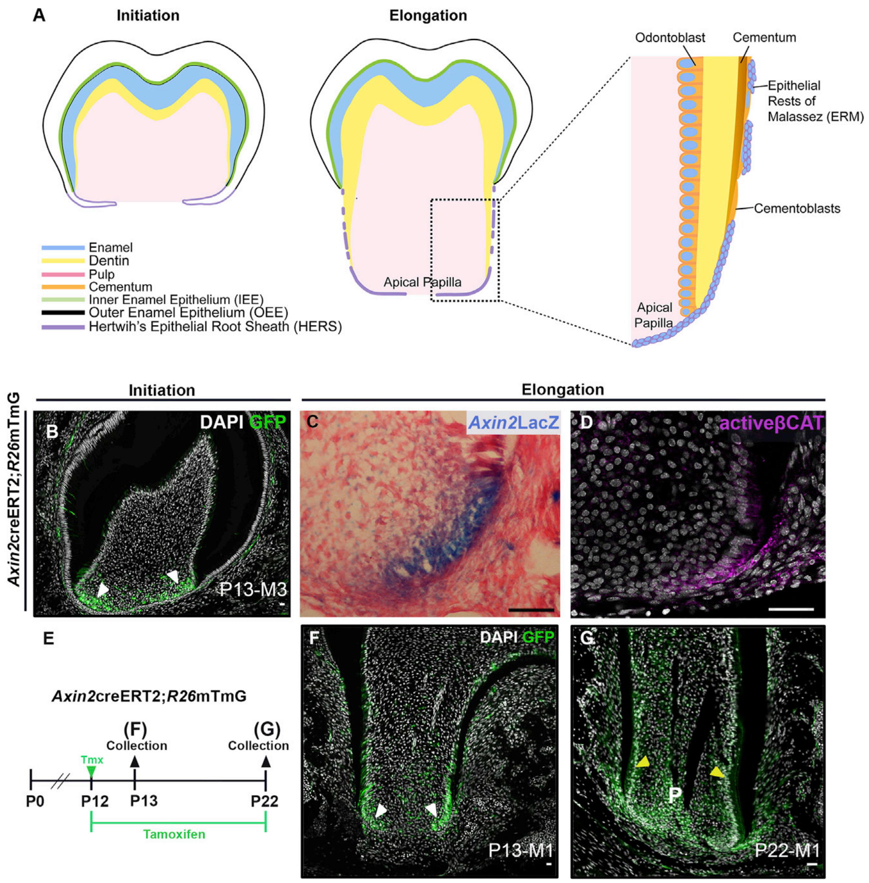

Figure 1. Wnt signaling is active during postnatal tooth development

(A) Morphodifferentiation during initiation and elongation stages of root formation.

(B) At initiation, Wnt responding cells (GFP) are found at the base of the crown (white arrowhead) of the developing third molar (M3) at P13.

(C) *Axin2*LacZ reporter mice show Wnt responding cells (blue) in the apical HERS and adjacent mesenchyme at P17 in M1.

(D) Activated β-CATENIN expression in epithelium and apical mesenchyme confirms canonical Wnt activity at P16 in M1.

(E) Tamoxifen (Tmx) time course administered to postnatal *Axin2*creERT2; R26mTmG mice for lineage tracing apical Wnt responding cells.

(F) During root elongation, Wnt responding cells are found at the apex of the forming root (white arrowhead) at P13 in M1.

(G) *Axin2* lineage cells form pulp (P) and odontogenic odontoblasts (yellow arrowhead) by P22. $n \geq 3$ tissue sections were examined from $n \geq 3$ mice.

Scale bars in (A)–(D), (F), and (G): 100 μm.

See also Figure S1.

development, the possible conserved relationships between Wnt action in the gut and tooth root was explored.

Here, we highlight the key role of the Wnt signaling pathway in the maintenance of the dental apical stem/progenitor population and in control of the integrity of the surrounding epithelial sheath. The findings demonstrate the conserved use of *Gli1*, *Sox9*, and Wnts to regulate stem/progenitor cell niches in both the tooth and gut, two distinct and highly specialized organ systems.

RESULTS

Wnt signaling is active in apical root stem/progenitor cells

To chart canonical Wnt activity during root development, *Axin2LacZ* reporter mice and inducible *Axin2*creERT2; R26mTmG mice were investigated. *Axin2* is a direct intracellular target of Wnt; therefore, *Axin2* reporter mice have been shown to reliably label Wnt responding cells (Lustig et al., 2002). At root initiation, *Axin2*$^{+}$ cells were located at the base of the crown (Figure 1B). During root elongation, these cells were localized to the apex of the molar root, at the advancing developmental front (Figures 1C and 1F). Wnt responding cells were found in both the apical HERS and adjacent mesenchymal cells, confirmed by the presence of activated β-CATENIN (Figure 1D).

The apex of the developing tooth root has previously been shown to house stem/progenitor cells that form the tooth root and its surrounding supporting structures (Sonoyama et al., 2008; Liu et al., 2015). Given the expression of *Axin2* in this region, the fate of the apical Wnt-responsive cells was followed using *Axin2-creERT2;R26mTmG* (*Axin2Lin*) mice during the stages of root elongation, when radicular hard and soft tissues are rapidly developing. Tamoxifen was administered to *Axin2-Lin* mice at P12 (postnatal day 12) and was traced for 10 days (until P22) (Figure 1E). The majority of root-forming cells giving rise to the dental pulp at the core, as well as dentinogenic odontoblasts at the periphery, were found to be derivatives of the initial apical Wnt-responsive stem/progenitor cell population, along with a subset of cells in the surrounding periodontium (Figures 1F and 1G).

The contribution of the *Axin2* lineage was compared in the incisors of P12 tamoxifen-injected *Axin2Lin* pups. The mouse incisor lacks a well-defined root and has a crown

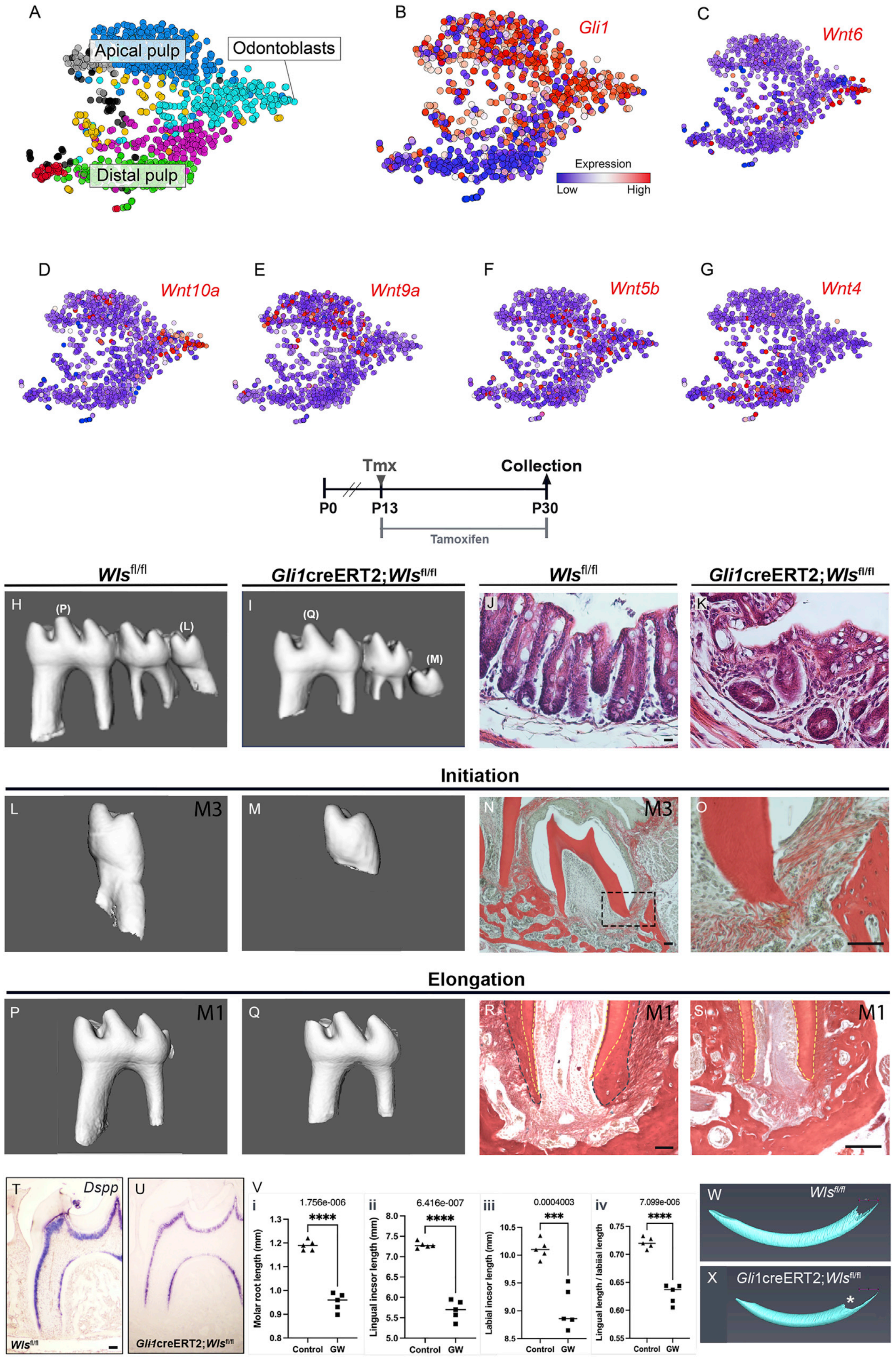

(legend on next page)

and root analog—the labial and lingual cervical loops, respectively, which, along with the proximal mesenchyme, house the stem cells that support its continuous growth (Renvoisé and Michon, 2014). At the beginning of the chase, *Axin2*-expressing Wnt responding cells were sparsely interspersed in mesenchymal tissue of the proximal pulp with a greater density adjacent to the lingual cervical loop. After 10 days of tamoxifen injection, *Axin2* lineage cells formed the bulk of the proximal pulp, including dentinogenic odontoblasts, and surrounding periodontal tissues (Figure S1). In contrast to molars, *Axin2*-expressing cells and their progeny had no significant contribution to the epithelial cervical loops (Figure S1C).

Wnts produced by *Gli1*$^{+}$ stem/progenitor cells are necessary for root development

Having charted Wnt activity, the source of the Wnt ligands in developing roots was traced. In the developing root, *Gli1* broadly labels a proliferative stem/progenitor population that gives rise to diverse cell lineages forming roots and periodontium (Figure S2). The *Axin2*-expressing stem/progenitor cells formed a subset of the broader *Gli1* population at the advancing front of developing roots (compare Figures 1C and S2A′). Given this close association, it was hypothesized that the *Gli1* population could be a crucial source of Wnt ligands. Analysis of a publicly available single-cell RNA sequencing (scRNA-seq) dataset generated from the murine incisor mesenchyme highlighted several Wnt ligands expressed by the *Gli1* dental population (Figures 2A and 2B) (Krivanek et al., 2020). Of particular note, *Wnt10a* and *Wnt9a* (both reported to act as canonical Wnts) were expressed in the apical pulp, along with *Wnt5b* (a non-canonical Wnt), while *Wnt6* was more closely associated with the odontoblast lineage, and *Wnt4* was expressed in the *Gli1*$^{-}$ distal pulp (Figures 2C–2G). To test the effect of loss of Wnts during root development, a loss-of- function transgenic murine model was utilized to specifically target *Wntless* production in *Gli1*-expressing cells (*Gli1*$^{creERT2/+}$;*Wls*$^{tm1.1Lan}$, hereafter referred to as *Gli1*creERT2;*Wls*$^{fl/fl}$). Abrogation of *Wntless* affects both canonical and non-canonical pathways (Bänziger et al., 2006) and would be predicted to lead to loss of WNT10a, -9a, -6, and -5b. Confirming the loss of canonical Wnt activity in the root, *Axin2* expression was specifically lost in the apical region (Figures S3A and S3B). Interestingly, *Axin2* was still robustly expressed in the periodontium, suggesting that there is another source of Wnt ligands in this region of the tooth (Figure S3C). In keeping with loss of Wnts from *Gli1*$^{+}$ cells, the mutant mice also showed gut defects, mimicking previously reported findings (Degirmenci et al., 2018) (Figures 2J and 2K). This deletion arrested root formation from the point of tamoxifen injection (Figures 2H and 2I). Loss of Wnt signaling at the initiation of root formation resulted in rootless teeth (Figures 2L–2O). In keeping with the presence of *Axin2*, the surrounding periodontal ligament (PDL) still formed, with fibers surrounding the unerupted, rootless molar

Figure 2. Wnts produced by *Gli1*$^{+}$ stem/progenitor cells are necessary for root formation
(A) Incisor pulp scRNA-seq data generated using smartseq2.
(B) The pulp is divided into an apical *Gli1* population and a distal non-*Gli1* population.
(C–G) Expression of Wnt ligands in the dental mesenchyme.
(H and I) Molar roots stop forming following abrogation of *Wntless* in *Gli1*$^{+}$ stem/progenitor cells, resulting in short-rooted mutant molars.
(H) Wild-type (WT) and (I) mutant microCT.
(J and K) Colon morphology was disrupted in mutants (K) compared with control mice (J).
(L–O) M3.
(L) WT microCT.
(M) Mutant microCT with no root.
(N and O) M1.
(P) WT microCT.
(Q) Mutant microCT with short root.
(R) Histology WT M1.
(S) Mutant histology. *Gli1*creERT2;*Wls*$^{fl/fl}$ mutants had blunt dentin stumps (yellow dashed line), resulting in a wide apex devoid of cementum (blue dashed line).
(T and U) *Dspp in situ* expression indicates normal differentiation of dentin-forming odontoblasts for given root length in control (T) and mutant teeth (U).
(V) The difference in M1 molar root length and the lingual and labial incisor length in control and mutant teeth was significant. Unpaired t-test. (i, ii & iv) $P < 0.0001$. (iii) $P < 0.0004$.
(W and X) MicroCT. Mutant mice exhibited shorter incisors affecting mainly the lingual side (root analog: white asterisk) of the proximal incisor (X) compared with littermate controls (W). n = 5 mice for mutant (*Gli1*creERT2;*Wls*$^{fl/fl}$) and control (non-Cre littermate) genotypes.
Scale bars in (J), (N), (O), and (R)–(T): 100 μm. Same scale in (K) as (J).
See also Figures S2–S5.

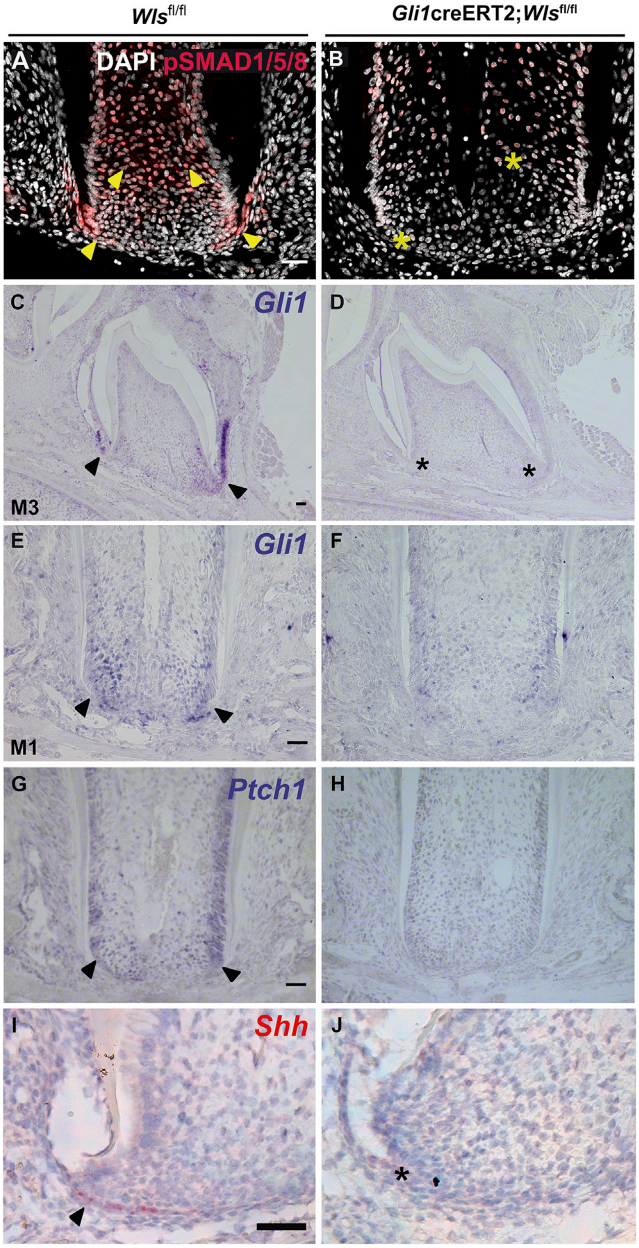

Figure 3. Wnts orchestrate apical signaling in the developing root

(A) BMP activity indicated by pSMAD expression (red) in the apical HERS and mesenchymal/pulp cells (yellow arrowhead) in littermate non-Cre controls was lost (yellow asterisk) in mutants with *Wls* deletion in *Gli1*$^{+}$ cells (B). *Gli1* and *Ptch1* mRNA (blue) expression (black arrowhead) in the apical epithelium and mesenchyme of developing molars (C, E, and G) was downregulated (black asterisk) in mutant molars (D, F, and H). *Shh* (red) normally expressed in the apical HERS (I) was also lost in mutant molars (J). $n \geq 3$ tissue sections were examined from $n \geq 3$ mice from mutant and control samples. Scale bars in (A), (C), (E), (G), and (I): 100 μm. Same scale in (B), (D), (F), (H), and (J).

(Figure 2O). Deletion at the stage of root elongation caused a significant reduction in tooth root length (Figure 2Vi) with wide, immature apices and defective cementum deposition (Figures 2P–2S). These short-rooted teeth erupted into the oral cavity and reached occlusion, in contrast to rootless third molars, which failed to erupt (Figures S3F and S3G). *In situ* staining for *Dspp*, a marker of odontoblasts, showed well-differentiated dentin-producing cells (Figures 2T and 2U). Our mutation, therefore, appears to specifically target radicular progenitors causing truncated root formation from the time the deletion is induced.

The impact of the deletion was also evident in the calvarium in regions adjacent to the cranial sutures (Figures S3D and S3E), indicating that *Gli1* progeny originating from cranial sutures (Zhao et al., 2015) also produce essential Wnts. By P30, the alveolar bone surrounding the tooth was brittle and irregularly deposited compared with littermate controls (Figures 2R and 2S). Inactivation of Wntless in developing bone has previously been shown to increase osteoclast activity (Zhong et al., 2012). Bone defects might, therefore, impact root formation. To investigate this, roots were analyzed at P18 after induction at P13, before any overt bone defects. In these mice, the roots were still shorter, and no difference in osteoclast activity was evident (Figures S4A–S4H). The short root phenotype therefore predates any later bone defects.

In keeping with the formation of truncated roots, loss of apical Wnts produced by *Gli1*$^{+}$ cells resulted in an almost complete loss of proliferation of apical cells in the root mesenchyme and epithelium (Figures S5A–S5D′), while the surrounding forming periodontium appeared to be largely unaffected (Figures S5B′ and S5D′).

Agreeing with the role of Wnts in the molar root, mutants exhibited overall shorter incisors (Figures 2Vii–2Viii, 2W, and 2X; Figures S3H and S3I), with a relatively greater disruption to the lingual cervical loop, which is known as the root analog (Figure 2Viv).

Loss of Wnt signaling disrupts signaling networks in the apical papilla

The BMP signaling cascade is responsible for regulation of cell proliferation and differentiation in many tissues, including skeletal hard tissues (Beederman et al., 2013). In the tooth root, activation of BMP signaling is required for the differentiation of dentin-forming odontoblasts (Feng et al., 2017). During normal root development, pSMAD1,5,9, a readout of BMP signaling, was observed in the apical HERS and was widespread in radicular mesenchymal tissue, barring the apical papilla (Figure 3A). Loss of apical Wnts produced by *Gli1*$^{+}$ cells resulted in a reduction in mesenchymal BMP activity and a complete loss

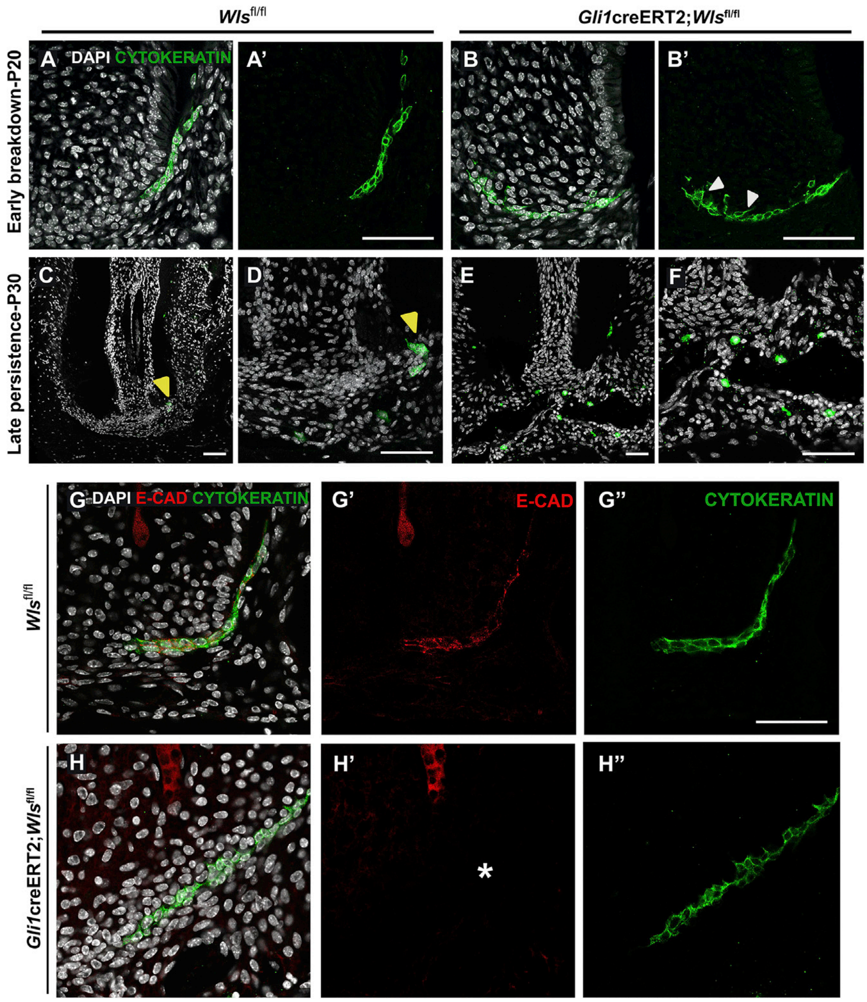

Figure 4. Loss of Wnt signaling in *Gli1*⁺ cells results in disruption to HERS

(A and A′) Bilayered low cuboidal HERS epithelial cells labeled with CYTOKERATIN (green) expression present an intact apical sheath during normal root development, which was disrupted within a week of *Wls* deletion in *Gli1*⁺ cells (B and B′). Disrupted sheath cells exhibit loss of cuboidal shape with abnormal cellular processes projecting from the cell body (white arrowhead).

(C and D, magnified) Following completion of root formation, rare remnants of sheath cells coalesce and persist as epithelial cell rests (yellow arrowhead) in the periodontium of control roots.

(E and F, magnified) Disrupted apical epithelial cells of mutant molars persist as numerous isolated cells interspersed within the apical root periodontium of the abnormal, short-rooted teeth even during late stages (P30).

(G–H″) The undisrupted apical epithelial root sheath cells (G, G′, and G″) are held together by E-CADHERIN (red), which is lost (white asterisk) in mutant molars (H, H′, and H″). n $\geq$ 3 tissue sections were examined from n $\geq$ 3 mice from mutant and control samples. Scale bar: 100 μm.

See also Figures S6A–S6D′.

within HERS (Figure 3B). Furthermore, members of the Shh signaling pathway were shown to be downregulated in the developing roots of mutant *Gli1*creERT2*;Wls*$^{fl/fl}$ molars (Figures 3C–3J), and *Gli1* expression was reduced in the mutant teeth (Figures 3C–3F). Reduced apical *Gli1* expression suggests potential autoregulation of these cells by the Wnt ligands they produce. Another target of the Shh pathway important for molar root development, *Ptch1* (Nakatomi et al., 2006), was also downregulated in mutant teeth (Figures 3G and 3H). *Shh* expression, which is normally found in the apical HERS (Nakatomi et al., 2006) and is crucial for maintaining epithelial-mesenchymal interaction at the root apex (Huang et al., 2009b), was also lost in mutant teeth (Figures 3I and 3J). These findings suggest that BMP and Shh signaling pathways are downstream targets under the control of Wnt signaling in apical stem/progenitor cells, although whether they are direct or indirect targets is unclear, as they may be consequences of the loss of specific populations.

Loss of apical Wnt ligands results in disruption to HERS

HERS shapes and guides root development (Fons Romero et al., 2017), and manipulating Wnt activity in HERS has an impact on the integrity of this important epithelial layer (Yang et al., 2021). The structure and gene expression of HERS was, therefore, investigated in the *Gli1*-creERT2;*Wls*$^{fl/fl}$ mice. Confirming the key role of Wnts in HERS, abrogation of *Wntless* resulted in a breakdown of epithelial integrity, with the formation of aberrant cellular projections evident a week after tamoxifen induction (Figures 4A–4B′). These projections were linked to a defect in the basement membrane surrounding HERS, demonstrated by loss of LAMININ staining (Figures S6A–S6D′). By P30, control root formation was complete, and most epithelial cells were lost, barring remnants that form the ERMs (Figures 4C and 4D). In *Gli1*creERT2*;Wls*$^{fl/fl}$ mutants, however, numerous persistent apical epithelial cells were found within the periodontal stroma (Figures 4E and 4F).

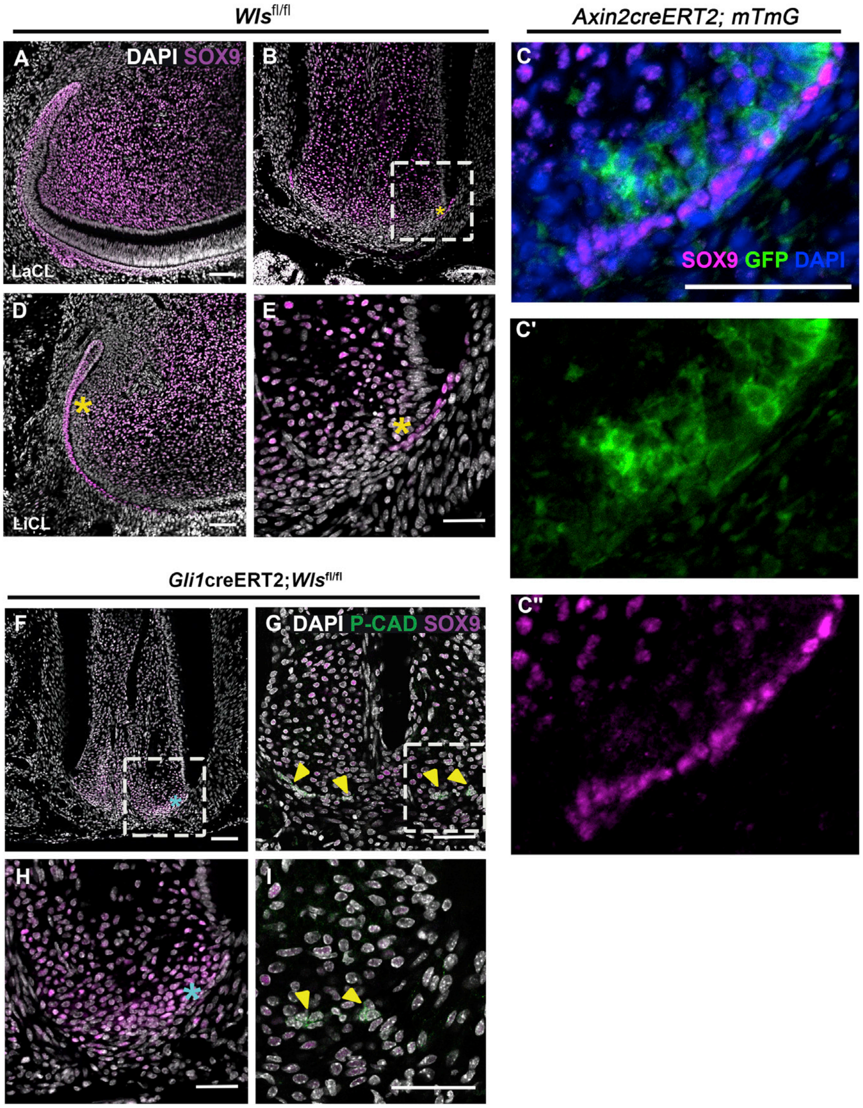

Figure 5. Loss of apical Wnts disregulates SOX9 expression in the developing molar

(A) In the mouse incisor, the juxta-epithelial mesenchymal cells of the labial cervical loop (crown analog) express SOX9 (pink), while (D) the lingual cervical loop (root analog) and molar (B and E, magnified) exhibit a narrow zone (yellow asterisk) of SOX9-devoid mesenchyme adjacent to the SOX9$^+$ apical HERS.

(C–C″) *Axin2*creERT2:*R26mTmG* reporter mice 1 day after Tmx injection (see schedule, Figure 1E) showing *Axin2* expression in green (GFP) (P13) (C and C′). Expression of *Axin2* is found in the apical region sandwiched between the SOX9-expressing mesenchyme and epithelium in pink (C and C‴).

(F and H) *Wls* deletion in *Gli1*$^+$ cells resulted in an overall downregulation of the broad mesenchymal SOX9-expressing domain (F and H, magnified) and upregulation of SOX9 in the normally SOX9-devoid apical zone (blue asterisk).

(G and I) Downregulation of SOX9 was noted within the fragmenting apical epithelium (yellow arrowhead) with concomitant loss of cell adhesion labeled with low P-CADHERIN (green) expression (G and I, magnified). $n \geq 3$ tissue sections were examined from $n \geq 3$ mice from mutant and control samples.

Scale bars in (A)–(I): 100 μm. Same scale in (C) as (C′) and (C″).

See also Figures S6E–S6H.

The change in HERS was accompanied by loss of E-CADHERIN expression, while the expression of pan-CYOKERATIN was maintained (Figures 4G–4H″). This suggests a convergent role of Wnts, potentially acting through β-catenin, a known component of the cadherin complex (Nelson and Nusse, 2004), thereby orchestrating cellular signaling while maintaining epithelial integrity.

Loss of apical Wnt signaling leads to misexpression of Sox9

Sox9 is a known marker of progenitor cells in numerous tissues, including hard tissues (Blache et al., 2004). In the gut, *Sox9*-expressing progenitor cells are essential for development and homeostasis and are regulated by Wnt ligands of epithelial and mesenchymal origin (Blache et al., 2004; Valenta et al., 2016). In teeth, *Sox9* labels progenitor cells in the enamel organ of the embryonic tooth germ (Kawasaki et al., 2015). In the continuously developing incisor, SOX9 expression is observed in the proximal epithelial and mesenchymal cells (Figures 5A and 5D), confirming previously reported homeostatic dental expression (Krivanek et al., 2020). Interestingly, a zone of mesenchyme devoid of SOX9 is located adjacent to the lingual cervical loop (root analog), while the juxta-epithelial mesenchymal cells of the labial cervical loop (crown analog) express SOX9 (Figures 5A and 5D). Much like the lingual cervical loop of the incisor, the molar root also exhibits a narrow zone of SOX9-devoid mesenchymal cells adjacent to HERS, sandwiched between the SOX9$^+$ epithelium and a broader SOX9$^+$ mesenchymal domain (Figures 5B and 5E). Interestingly, this SOX9$^-$ mesenchymal domain expresses *Axin2*, suggesting

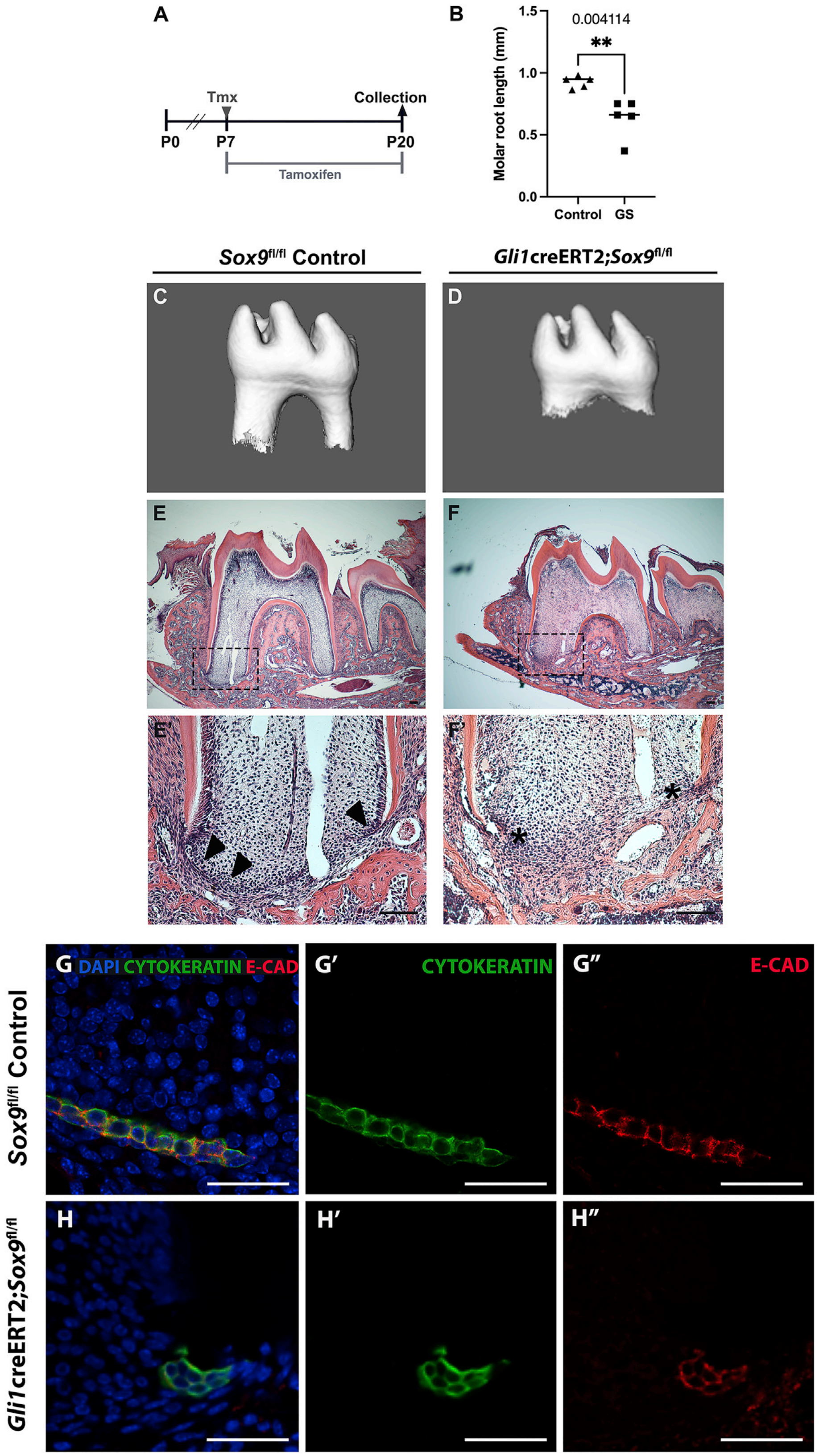

(legend on next page)

compartmentalization of the apical mesenchyme into *Axin2* and SOX9 domains (Figures 5C–5C″). Loss of Wnt ligand production in *Gli1*-expressing cells, and loss of the *Axin2* domain, resulted in expansion of SOX9 expression in the mesenchyme up to HERS (Figures 5F and 5H), suggesting that *Axin2* normally restricts the expression of SOX9 in the molar. In contrast to the expansion of SOX9 in the apical mesenchyme, SOX9 expression was lost within the apical epithelium of HERS (Figures 5G and 5I). Loss of Wnt signaling thus leads to loss of E-CADHERIN and SOX9 in the root epithelium (Figures S6E–S6H).

Sox9 expression in *Gli1*$^+$ stem/progenitor cells is essential for molar root development

Having observed a downregulation of SOX9 expression in the epithelium of *Gli1*creERT2;*Wls*$^{fl/fl}$ mutant roots, we then asked if apical *Sox9* expression is necessary for root formation. To investigate this, *Gli1*$^{creERT2/+}$; *Sox9*^{tm2Crm} (hereafter referred to as *Gli1*creERT2;*Sox9*$^{fl/fl}$) transgenic mice were generated. Loss of SOX9 in the forming roots was confirmed by immunofluorescence (Figures S7B and S7C), with the mutant mice displaying severe hair defects, as would be expected given the essential role of *Sox9* in the hair stem cell compartment (Figure S7A) (Vidal et al., 2005). To capture early effects on root formation while maintaining vitality of the experimental model, the mutation was induced during early postnatal life at P7 (Figure 6A). The molar roots of the *Gli1*creERT2;*Sox9*$^{fl/fl}$ mice were shorter than littermate controls (Figures 6B–6F), while the incisors appeared largely unaffected (Figures S7H–S7K). The apical HERS exhibited aberrant morphology, forming a clump of epithelial cells at the edges of the developing root instead of the bilayered sheath observed in control roots (Figures 6E′ and 6F′; Figures S7D–S7G), similar to the disruption in HERS in the *Gli1*creERT2;*Wls*$^{fl/fl}$ mutants. However, unlike the *Gli1*creERT2;*Wls*$^{fl/fl}$ HERS phenotype, *Gli1*creERT2;*Sox9*$^{fl/fl}$ mutant roots retained apical E-CADHERIN expression (Figures 6G–6H″). Overall, this indicates that *Sox9* is an essential transcription factor for molar root formation, playing an important role in HERS stability, although its abrogation produced a milder phenotype compared with the dramatic rootless teeth produced by loss of Wnt ligand production in *Gli1*$^+$ stem/progenitor cells.

Overexpression of Wnts in the developing root apex results in rapid differentiation of odontogenic precursor cells

Given the importance of Wnt for root development, the effect of Wnt overactivation during root formation was explored. In the gut, ectopic Wnt secretion disrupts crypt epithelial stem cells and results in increased instances of polyposis and neoplasia (Harada et al., 1999). To test the effect of Wnt overactivity in the developing root, a transgenic mouse line that conditionally expressed an activated form of *Ctnnb1* in *Axin2*-expressing cells, *Axin2*$^{creERT2/+}$;*R26*$^{mTmG/+}$; *Ctnnb1*$^{lox(ex3)/+}$, was generated. To target stages of active root formation, a Cre-driven gain-of-function mutation was conditionally induced by administering tamoxifen at P15. Tissues were harvested at P28 for phenotypic assessment and at P20 to investigate the cellular and molecular changes leading to the phenotypic changes (Figures 7A and 7G). In the developing root, stimulation of canonical Wnt signaling resulted in rapid differentiation of dental hard tissue-forming cells, both odontoblasts and cementoblasts (Figures 7B–7E and 7H–7I′). The resultant root surface was irregular with thickened apical margins (Figures 7C and 7E). The odontoblasts (especially in the apex) appeared to have lost their polarization, were more numerous, and laid down irregular masses of dentin, indicating rapid disorderly deposition (Figure 7I′). Isolated masses of dentinoid were observed within the pulp, suggestive of premature odontogenic differentiation of precursor/progenitors found in the apical dental pulp (Figure 7I′). The surrounding cementum was irregular and very cellular, reflective of the rapid rate of hard tissue deposition with increased cellular entrapment. Similar effects have been reported in murine gain-of-function models with targeted constitutive activation of β-CATENIN in odontoblasts (Wang et al., 2021). Interestingly, the apical HERS lost its

Figure 6. *Sox9* expression in *Gli1*$^+$ cells is essential for molar root development
(A) Tmx time course.
(B–H) Significant root length shortening (B) was caused by the deletion of *Sox9* in *Gli1*-expressing cells in mutant (*Gli1*creERT2;*Sox9*$^{fl/fl}$) mice (C and E) compared with littermate controls (D and F). n = 6 mice. Hematoxylin and eosin-stained histology sections (E and F) and immuno-expression of cytokeratin (green) used to label epithelial cells show clumping of epithelial cells at the edges of the developing mutant root (F and H) instead of the bilayered sheath seen in control roots (E and G). Clumped apical epithelial cells of the mutant teeth retain E-CADHERIN expression (red). Black arrowhead points to apical HERS, whereas black asterisk indicates anomalous shortening of HERS. $n \geq 3$ tissue sections were examined from $n \geq 3$ mice with mutant and control genotype.
(B) Unpaired t-test. $P < 0.004$. Scale bars in (E)–(H): 100 μm.
See also Figure S7.

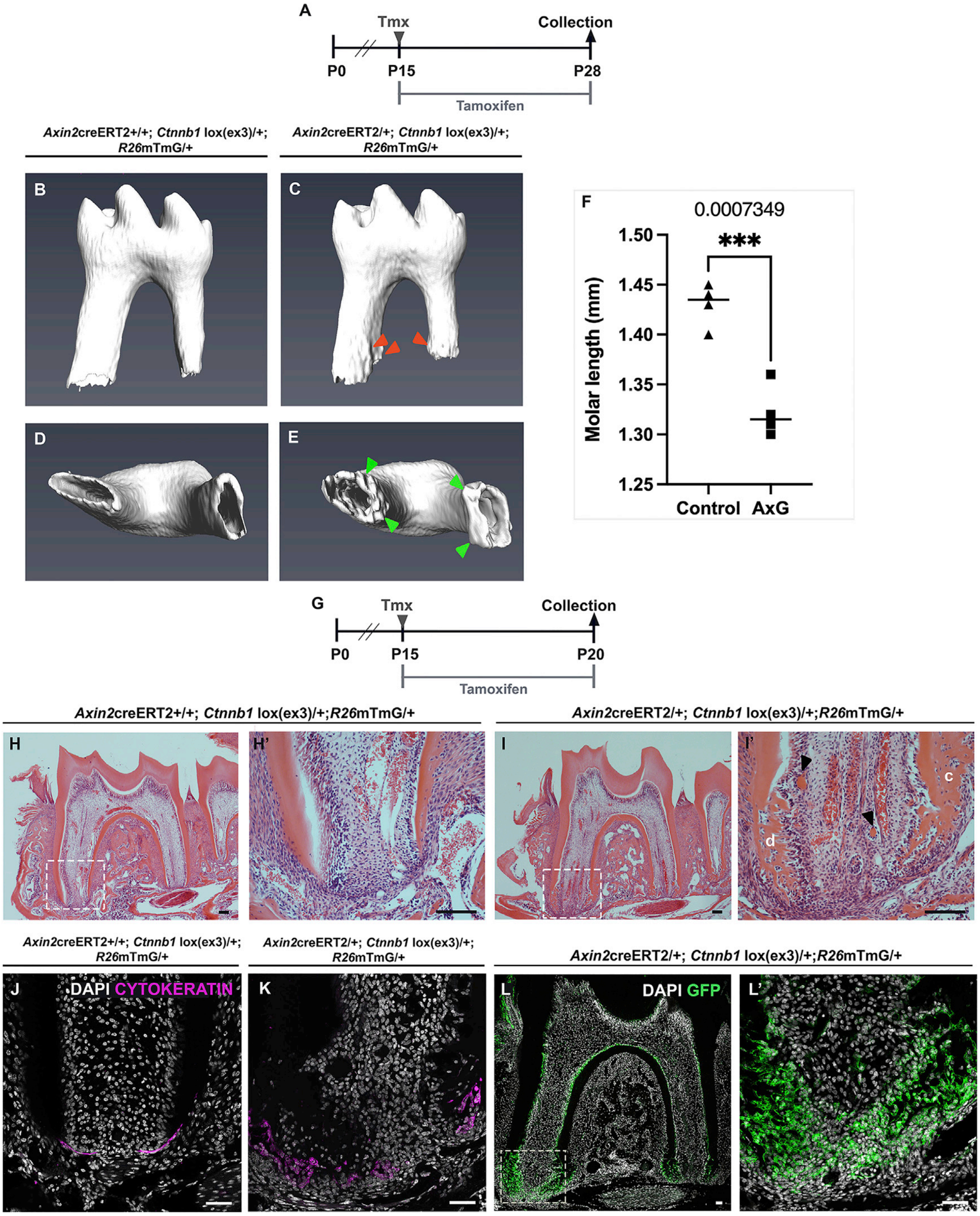

Figure 7. Overexpression of Wnts in the developing roots results in rapid differentiation of odontogenic precursor cells in the developing molar

(A) Tmx time course for microCT analysis.

(B–F) Compared with non-Cre littermate controls (B and D), constitutive stabilization of β-CATENIN in Wnt responding cells of the developing mutant molar root results in shorter roots (C, E, and F) with haphazard deposition of radicular hard tissue (orange arrowhead) and thickening of the apical rim (green arrowhead). n = 4 mice.

(G) Tmx time course for cellular and molecular investigation.

(legend continued on next page)

bilayer architecture (Figures 7J and 7K). This phenotype was a direct consequence of the genetic overactivation of the Wnt pathway, evidenced by the distribution of *Axin2* lineage cells specifically in the newly formed root following induction of the mutation (Figure 7L′).

DISCUSSION

Development and homeostasis utilize a conserved set of biochemical signaling pathways to activate intracellular transcriptional networks that guide cell proliferation, specification, and quiescence. Here, we examined the role of the Wnt signaling pathway in postnatal tooth development and found similarities in the way these developmental pathways and their intracellular transcriptional targets are used across two morphologically, functionally, and developmentally distinct organ systems—teeth and gut.

Stem/progenitor cells at the base of the developing molar root were shown to be Wnt-responsive *Axin2*-expressing epithelial and mesenchymal cells that gave rise to all dental hard and soft tissue-forming cells. *Axin2*, therefore, could be a marker for the root apical stem/progenitor population. This parallels the gut, where *Axin2*-labeled stem cells at the base of the epithelial crypts form all cell lineages of the intestinal crypts and villi (Flanagan et al., 2018; Harnack et al., 2019). Unlike the intestine, postnatal molar roots are primarily mesenchymal derivatives. Here, the epithelium guides root form (Fons Romero et al., 2017) and maintains intercellular signaling crosstalk with the underlying mesenchymal cells during development but then largely disappears (Huang et al., 2009a; Zeichner-David et al., 2003; Li et al., 2017). Similar to molars, *Axin2*$^{+}$ cells were found in the mesenchyme of the proximal incisors, which form the putative stem/progenitor cell compartment of this homeostatic model; however, unlike molars, the proximal epithelium was largely devoid of *Axin2*-expressing cells and their progeny.

Wnt ligands are short-range glycoproteins (Farin et al., 2016); hence, the sources of these ligands are usually positioned close to responding cells. This has been demonstrated in the murine colon (Valenta et al., 2016; Degirmenci et al., 2018). By abrogating Wnt ligand production specifically in the apical *Gli1*$^{+}$ stem/progenitor cells, we demonstrated that Wnt signaling is essential for postnatal dental development. The effect on the developing molar roots was stark, with cessation of root development upon abrogation of *Wntless* production. This is likely to involve both canonical and non-canonical Wnt activity, with both canonical and non-canonical Wnt ligands shown to be produced by the *Gli1* population. In this article, we have focused on *Axin2* as a canonical readout, but it would be interesting to explore the impact on non-canonical targets, which may be linked to the changes in the integrity of HERS. Of the Wnt ligands identified in the *Gli1* population, loss of *Wnt10a* is known to lead to root defects, with the formation of non-bifurcating taurodont teeth in mice and patients (Yang et al., 2015). The root cessation phenotype described here is more severe, representing the accumulated of loss of all Wnt ligands in the *Gli1* population. The relative roles of the other Wnts, such as *Wnt9a*, will be an interesting area for exploration in the future.

Since *Gli1* is a broad stem/progenitor cell marker in the developing root, abrogation of Wnt ligand production was achieved in both epithelial and mesenchymal apical cells. Therefore, it is unclear whether Wnts produced by epithelial or mesenchymal cells are independently sufficient for root development. The gut exhibits regional variability in its dependence on epithelial vs. mesenchymal Wnt ligands. Mesenchymal Wnts are indispensable in the colon, where their loss results in complete disintegration of the epithelium, while the duodenum is spared unless both sources are lost (Degirmenci et al., 2018; Valenta et al., 2016). Future investigations to establish the effect of epithelial and mesenchymal Wnt ligands on postnatal dental development will be useful. In the gut, *Sox9*-expressing progenitor cells are essential for development and homeostasis and are regulated by Wnt ligands (Blache et al., 2004; Valenta et al., 2016). Here, we show a similar essential role for *Sox9* in the developing tooth and a reliance of Wnt signaling for its epithelial expression. In the apical mesenchyme of the molar root, SOX9 was excluded from the *Axin2* expression domain. A similar absence of SOX9 in the *Axin2* domain was observed in the lingual loop of the incisor. In keeping with this, loss of *Axin2* in *Gli1*creERT2;*Wls*$^{fl/fl}$ mutants led to an expansion of SOX9 into this juxta-epithelial

(H–I′) Hematoxylin and eosin–stained histology sections show exuberant and irregular dentin (d) and cellular cementum (c) deposition in the mutant root (I and I′) compared with control roots (H and H′). Isolated dentinoid islands (black arrowhead) interspersed within the pulp of mutant root (I′).
(J and K) Cytokeratin (pink) expression in the mutant root shows prolific apical epithelium with loss of bilayered architecture (K), otherwise seen in the control root (J).
(L and L′) GFP (green) immuno-expression labels *Axin2* lineage cells seen in abundance at the apex of the irregularly developing mutant root. n = 3 mice with mutant (*Axin2*$^{creERT2/+}$;*R26*$^{mTmG/+}$; *Ctnnb1*$^{lox(ex3)/+}$) and control (non-Cre littermates) genotypes.
(F) Unpaired t-test. P < 0.0007. Scale bars in (H)–(L): 100 μm.

mesenchymal domain. Regulation of SOX9 in the molar epithelium and mesenchyme is, therefore, distinct with potential opposing roles of Wnt signaling.

In developing molar roots, the *Gli1*creERT2;*Wls*$^{fl/fl}$ loss-of-function mutation selectively hampered radicular progenitors, while the periodontal progenitors were spared. This is particularly interesting since both these progenitor cell populations arise from the same apical *Gli1*-expressing stem cell domain (Men et al., 2020). Our findings indicate a reliance of the periodontium on Wnt ligands from alternate sources, highlighted by the persistence of *Axin2* expression and proliferation in this region. Wnt ligands may therefore originate from part of the larger *Thy1*-expressing progenitor cell population (Zhao et al., 2021).

Differential regulation of dental progenitor cells was also observed between developing molars and homeostatic incisors and between the labial and lingual cervical loops within the proximal incisor when Wnt ligand production by *Gli1*-expressing cells was lost. The different outcomes observed on the labial and lingual side of the mutant incisors were interesting since *Gli1* labels proximal epithelial and mesenchymal stem cells across both cervical loops of the murine incisor. These findings highlight the molecular similarities between the molar root and the incisor lingual cervical loop, supporting the classification of the lingual loop as a root analog.

Similar to the gut and ectodermal organs such hair and salivary glands, tooth development is guided by interactions between the key developmental signaling pathways. Both gut and teeth have Wnts at the helm, maintaining the stem/progenitor cell pool and interacting with SHH, transforming growth factor β (TGF-β), and BMP signaling pathways, guiding proliferation and differentiation. Here, we demonstrated how derangement in Wnt signaling led to lowered SHH and BMP signaling, although it is unclear whether this is through a direct or indirect mechanism. Co-localization of *Axin2*, *Gli1*, *Shh*, and activated pSMAD1,5,8 in the apical HERS suggests that this bilayered epithelial sheath is the core signaling center where the Wnt, SHH, and BMP signaling pathways act in parallel and perhaps converge to regulate root formation.

This study highlights the conserved nature of molecular interactions across two distinct tissue types—tooth and gut. Our results demonstrate how lessons in development and homeostasis can provide potential clues about cellular interactions occurring during development and homeostasis in health and disease across distinct tissues. Furthermore, this new understanding lends further support to the vital role of Wnts in the maintenance of the tooth stem/progenitor cell niche and radicular hard tissue formation and provides new insight into the diverse mechanisms of their action. A clearer understanding of the molecular mechanisms of stem/progenitor cell behavior and regulation during development will help us decipher the etiopathogenesis of developmental anomalies and disease states, which will form the basis for novel precision diagnostic and therapeutic modalities.

EXPERIMENTAL PROCEDURES

Resource availability

Corresponding author

Further requests for resources and reagents should be directed to and will be fulfilled by the corresponding author, Abigail Tucker (Abigail.tucker@kcl.ac.uk).

Materials availability

The study did not generate new unique reagents.

Data and code availability

Data will be shared with the research community upon request to the authors. No code or standardized datasets were generated.

Murine models

Transgenic (maintained on a mixed background) and wild-type mice used in this investigation were housed in controlled conditions at the King's College London Biological Services Unit. Wild-type CD1 mice were obtained from CRL (Charles River Laboratory, Edinburgh, UK) and transgenic mouse lines *Axin2*creERT2 (Lustig et al., 2002); *Axin2*LacZ (Lustig et al., 2002); *Gli1*creERT2 (Ahn and Joyner, 2004); R26R-mTmG (Muzumdar et al., 2007); R26R-tdTomato (Madisen et al., 2010); *Wls*$^{fl/fl}$ (Carpenter et al., 2010); *Sox9*$^{fl/fl}$ (Kist et al., 2002); and *Ctnnb1*$^{lox(ex3)/+}$ (Harada et al., 1999) have been previously described. Postnatal mice were culled following schedule-one methods as approved by the UK Home Office and were performed by trained individuals. Use of genetically modified mice was approved by the local GMO committee at King's, under personal and project licenses in accordance with the Animal (Scientific Procedures) Act of 1986, UK. This project conforms with the ARRIVE (Animal Research: Reporting of *In Vivo* Experiments) guidelines.

Immunofluorescence

Sections were deparaffinized and rehydrated. Heat-induced antigen retrieval was carried out in 0.1 M sodium citrate (pH 6) buffer. Tissue sections were then blocked in 1% BSA, 0.1% Triton-X, and 1% serum. Sections were then treated with primary antibodies overnight at 4°C. For BrdU immunofluorescence, sections were additionally treated for 30 min with 2 M HCl at 40°C before incubation with primary antibody. After secondary antibodies, slides were mounted with Fluoroshield containing DAPI (Abcam). Sections were imaged with a Leica TCS SP5 confocal microscope. To ensure true and specific protein expression, positive and negative (without primary antibodies) procedural controls were maintained. Images were processed using Fiji (Schindelin et al., 2012). See supplemental experimental methods for antibody details.

Tartrate-resistant acid phosphatase (TRAP) staining was carried out as described in Grigoriadis et al. (1994).

In situ hybridization and RNAscope

Dig-labeled antisense RNA probes were synthesized against mouse *Gli1* and *Ptch1* mRNA, and *in situ* hybridization was carried out to detect the expression in sagittal sections of wild-type mice, as previously described (Fons Romero et al., 2017). For the analysis of *Axin2*, RNAscope multiplex fluorescence assay (400,331-C3) (Advance Cell Diagnostics [ACD], a BioTechne brand) was performed following the manufacturer's instructions.

MicroCT and 3D reconstruction

PFA fixed mouse heads (n $\geq$ 5 samples each of mutant and control genotype) were dissected and scanned with a GE Locus SP microcomputed tomography (microCT) scanner and a Scanco microCT 50 scanner. 3D reconstructions and hard tissue image analysis of molars, incisors, and whole head were generated using MicroView and Amira. Statistics involved non-paired t tests to compare mutant vs. wild-type samples.

Transcriptomics

Publicly available scRNA-seq data generated from the mouse incisor pulp using smarseq2 were analyzed to assess the Wnt ligands present in the Gli1/apical mesenchyme (Krivanek et al., 2020) (http://pklab.med.harvard.edu/ruslan/dental.atlas.html).

SUPPLEMENTAL INFORMATION

Supplemental information can be found online at https://doi.org/10.1016/j.stemcr.2023.02.004.

AUTHOR CONTRIBUTIONS

R.L. and A.S.T. conceptualized and designed the study. R.L. and J.K. collected data. R.L., N.A., and A.S.T. analyzed and interpreted data. R.L., N.A., and A.T. drafted and revised the manuscript. All authors critically revised the manuscript and approved the final version.

ACKNOWLEDGMENTS

We thank Christopher Healy for help with microCT scanning and Marcos Gonzalez-Lopez for help with RNAscope. Imaging and processing of hard and soft tissue samples were carried out at King's College London. The project was funded by a Project grant from the BBSRC (BB/W00240X/1). R.L. was additionally funded by the King's International scholarship scheme. The research of J.K. was supported by Czech Science Foundation grant no. 23-06160S.

CONFLICT OF INTERESTS

A.S.T. is also affiliated with Charles University, Czech Republic.

Received: July 16, 2022
Revised: February 15, 2023
Accepted: February 15, 2023
Published: March 16, 2023

REFERENCES

Ahn, S., and Joyner, A.L. (2004). Dynamic changes in the response of cells to positive hedgehog signaling during mouse limb patterning. Cell *118*, 505–516. https://doi.org/10.1016/j.cell.2004.07.023.

Bae, C.H., Kim, T.H., Ko, S.O., Lee, J.C., Yang, X., and Cho, E.S. (2015). Wntless regulates dentin apposition and root elongation in the mandibular molar. J. Dent. Res. *94*, 439–445. https://doi.org/10.1177/0022034514567198.

Bänziger, C., Soldini, D., Schütt, C., Zipperlen, P., Hausmann, G., and Basler, K. (2006). Wntless, a conserved membrane protein dedicated to the secretion of Wnt proteins from signaling cells. Cell *125*, 509–522. https://doi.org/10.1016/j.cell.2006.02.049.

Barker, N., Rookmaaker, M.B., Kujala, P., Ng, A., Leushacke, M., Snippert, H., van deWetering, M., Tan, S., Van Es, J.H., Huch, M., et al. (2012). Lgr5+ve stem/progenitor cells contribute to nephron formation during kidney development. Cell Rep. *2*, 540–552. https://doi.org/10.1016/j.celrep.2012.08.018.

Beederman, M., Lamplot, J.D., Nan, G., Wang, J., Liu, X., Yin, L., Li, R., Shui, W., Zhang, H., Kim, S.H., et al. (2013). BMP signaling in mesenchymal stem cell differentiation and bone formation. J. Biomed. Sci. Eng. *6*, 32–52. https://doi.org/10.4236/jbise.2013.68A1004.

Blache, P., van de Wetering, M., Duluc, I., Domon, C., Berta, P., Freund, J.-N., Clevers, H., and Jay, P. (2004). SOX9 is an intestine crypt transcription factor, is regulated by the Wnt pathway, and represses the CDX2 and MUC2 genes. J. Cell Biol. *166*, 37–47. https://doi.org/10.1083/jcb.200311021.

Carpenter, A.C., Rao, S., Wells, J.M., Campbell, K., and Lang, R.A. (2010). Generation of mice with a conditional null allele for wntless. Genesis *48*, 554–558. https://doi.org/10.1002/dvg.20651.

Clevers, H., Loh, K.M., and Nusse, R. (2014). An integral program for tissue renewal and regeneration: Wnt signaling and stem cell control. Science *346*, 1248012. https://doi.org/10.1126/science.1248012.

Degirmenci, B., Valenta, T., Dimitrieva, S., Hausmann, G., and Basler, K. (2018). GLI1-expressing mesenchymal cells form the essential Wnt-secreting niche for colon stem cells. Nature *558*, 449–453. https://doi.org/10.1038/s41586-018-0190-3.

Farin, H.F., Jordens, I., Mosa, M.H., Basak, O., Korving, J., Tauriello, D.V.F., de Punder, K., Angers, S., Peters, P.J., Maurice, M.M., and Clevers, H. (2016). Visualization of a short-range Wnt gradient in the intestinal stem-cell niche. Nature *530*, 340–343. https://doi.org/10.1038/nature16937.

Feng, J., Jing, J., Li, J., Zhao, H., Punj, V., Zhang, T., Xu, J., and Chai, Y. (2017). BMP signaling orchestrates a transcriptional network to control the fate of mesenchymal stem cells in mice. Development *144*, 2560–2569. https://doi.org/10.1242/dev.150136.

Flanagan, D., Austin, C., Vincan, E., and Phesse, T. (2018). Wnt Signalling in Gastrointestinal Epithelial Stem Cells. Genes *9*, 178. https://doi.org/10.3390/genes9040178.

Fons Romero, J.M., Star, H., Lav, R., Watkins, S., Harrison, M., Hovorakova, M., Headon, D., and Tucker, A.S. (2017). The impact of the eda pathway on tooth root development. J. Dent. Res. *96*, 1290–1297. https://doi.org/10.1177/0022034517725692.

Grigoriadis, A.E., Wang, Z.,Q., Cecchini, M.G., Hofstetter, W., Felix, R., Fleisch, H.A., and Wagner, E.F. (1994). C-fos: a key regulator

of osteoclast-macrophage lineage determination and bone remodelling. Science *266*, 443–448.

Harada, N., Tamai, Y., Ishikawa, T., Sauer, B., Takaku, K., Oshima, M., and Taketo, M.M. (1999). Intestinal polyposis in mice with a dominant stable mutation of the beta-catenin gene. EMBO J. *18*, 5931–5942. https://doi.org/10.1093/emboj/18.21.5931.

Harnack, C., Berger, H., Antanaviciute, A., Vidal, R., Sauer, S., Simmons, A., Meyer, T.F., and Sigal, M. (2019). R-spondin 3 promotes stem cell recovery and epithelial regeneration in the colon. Nat. Commun. *10*, 4368. https://doi.org/10.1038/s41467-019-12349-5.

Huang, X., Bringas, P., Slavkin, H.C., and Chai, Y. (2009a). Fate of HERS during tooth root development. Dev. Biol. *334*, 22–30. https://doi.org/10.1016/j.ydbio.2009.06.034.

Huang, X., Xu, X., Bringas, P., Hung, Y.P., and Chai, Y. (2010). Smad4-Shh-Nfic signaling cascade-mediated epithelial-mesenchymal interaction is crucial in regulating tooth root development. J. Bone Miner. Res. *25*, 1167–1178. https://doi.org/10.1359/jbmr.091103.

Jussila, M., and Thesleff, I. (2012). Signaling networks regulating tooth organogenesis and regeneration, and the specification of dental mesenchymal and epithelial cell lineages. Cold Spring Harb. Perspect. Biol. *4*, a008425. https://doi.org/10.1101/cshperspect.a008425.

Kawasaki, K., Kawasaki, M., Watanabe, M., Idrus, E., Nagai, T., Oommen, S., Maeda, T., Hagiwara, N., Que, J., Sharpe, P.T., and Ohazama, A. (2015). Expression of Sox genes in tooth development. Int. J. Dev. Biol. *59*, 471–478. https://doi.org/10.1387/ijdb.150192ao.

Kim, T.H., Bae, C.H., Lee, J.C., Ko, S.O., Yang, X., Jiang, R., and Cho, E.S. (2013). β-Catenin is required in odontoblasts for tooth root formation. J. Dent. Res. *92*, 215–221. https://doi.org/10.1177/0022034512470137.

Kist, R., Schrewe, H., Balling, R., and Scherer, G. (2002). Conditional inactivation of Sox9: a mouse model for campomelic dysplasia. Genesis *32*, 121–123. https://doi.org/10.1002/gene.10050.

Kramann, R., Goettsch, C., Wongboonsin, J., Iwata, H., Schneider, R.K., Kuppe, C., Kaesler, N., Chang-Panesso, M., Machado, F.G., Gratwohl, S., et al. (2016). Adventitial MSC-like cells are progenitors of vascular smooth muscle cells and drive vascular calcification in chronic kidney disease. Cell Stem Cell *19*, 628–642. https://doi.org/10.1016/j.stem.2016.08.001.

Krivanek, J., Soldatov, R.A., Kastriti, M.E., Chontorotzea, T., Herdina, A.N., Petersen, J., Szarowska, B., Landova, M., Matejova, V.K., Holla, L.I., et al. (2020). Dental cell type atlas reveals stem and differentiated cell types in mouse and human teeth. Nat. Commun. *11*, 4816. https://doi.org/10.1038/s41467-020-18512-7.

Li, J., Parada, C., and Chai, Y. (2017). Cellular and molecular mechanisms of tooth root development. Development *144*, 374–384. https://doi.org/10.1242/dev.137216.

Liu, G., Vijayakumar, S., Grumolato, L., Arroyave, R., Qiao, H., Akiri, G., and Aaronson, S.A. (2009). Canonical Wnts function as potent regulators of osteogenesis by human mesenchymal stem cells. J. Cell Biol. *185*, 67–75. https://doi.org/10.1083/jcb.200810137.

Liu, Y., Feng, J., Li, J., Zhao, H., Ho, T.-V., and Chai, Y. (2015). An Nfic-hedgehog signaling cascade regulates tooth root development. Development, 127068. https://doi.org/10.1242/dev.127068.

Lohi, M., Tucker, A.S., and Sharpe, P.T. (2010). Expression of Axin2 indicates a role for canonical Wnt signaling in development of the crown and root during pre- and postnatal tooth development. Dev. Dyn. *239*, 160–167. https://doi.org/10.1002/dvdy.22047.

Lustig, B., Jerchow, B., Sachs, M., Weiler, S., Pietsch, T., Karsten, U., van de Wetering, M., Clevers, H., Schlag, P.M., Birchmeier, W., and Behrens, J. (2002). Negative feedback loop of Wnt signaling through upregulation of conductin/axin2 in colorectal and liver tumors. Mol. Cell Biol. *22*, 1184–1193. https://doi.org/10.1128/MCB.22.4.1184-1193.2002.

Ma, Y., Jing, J., Feng, J., Yuan, Y., Wen, Q., Han, X., He, J., Chen, S., Ho, T.-V., and Chai, Y. (2020). Ror2-mediated non-canonical wnt signaling regulates Cdc42 and cell proliferation during tooth root development. Development, 196360. https://doi.org/10.1242/dev.196360.

Madisen, L., Zwingman, T.A., Sunkin, S.M., Oh, S.W., Zariwala, H.A., Gu, H., Ng, L.L., Palmiter, R.D., Hawrylycz, M.J., Jones, A.R., et al. (2010). A robust and high-throughput Cre reporting and characterization system for the whole mouse brain. Nat. Neurosci. *13*, 133–140. https://doi.org/10.1038/nn.2467.

Men, Y., Wang, Y., Yi, Y., Jing, D., Luo, W., Shen, B., Stenberg, W., Chai, Y., Ge, W.-P., Feng, J.Q., and Zhao, H. (2020). Gli1+ periodontium stem cells are regulated by osteocytes and occlusal force. Dev. Cell *54*, 639–654.e6. https://doi.org/10.1016/j.devcel.2020.06.006.

Muzumdar, M.D., Tasic, B., Miyamichi, K., Li, L., and Luo, L. (2007). A global double-fluorescent Cre reporter mouse. Genesis *45*, 593–605. https://doi.org/10.1002/dvg.20335.

Nakatomi, M., Morita, I., Eto, K., and Ota, M.S. (2006). Sonic hedgehog signaling is important in tooth root development. J. Dent. Res. *85*, 427–431. https://doi.org/10.1177/154405910608500506.

Nelson, W.J., and Nusse, R. (2004). Convergence of Wnt, beta-catenin, and cadherin pathways. Science *303*, 1483–1487. https://doi.org/10.1126/science.1094291.

Oka, K., Morokuma, M., Imanaka-Yoshida, K., Sawa, Y., Isokawa, K., and Honda, M.J. (2012). Cellular turnover in epithelial rests of Malassez in the periodontal ligament of the mouse molar. Eur. J. Oral Sci. *120*, 484–494. https://doi.org/10.1111/eos.12003.

Plaks, V., Brenot, A., Lawson, D.A., Linnemann, J.R., Van Kappel, E.C., Wong, K.C., de Sauvage, F., Klein, O.D., and Werb, Z. (2013). Lgr5-Expressing cells are sufficient and necessary for postnatal mammary gland organogenesis. Cell Rep. *3*, 70–78. https://doi.org/10.1016/j.celrep.2012.12.017.

Renvoisé, E., and Michon, F. (2014). An Evo-Devo perspective on ever-growing teeth in mammals and dental stem cell maintenance. Front. Physiol. *5*, 324. https://doi.org/10.3389/fphys.2014.00324.

Sarkar, L., and Sharpe, P.T. (1999). Expression of Wnt signalling pathway genes during tooth development. Mech. Dev. *85*, 197–200. https://doi.org/10.1016/S0925-4773(99)00095-7.

Schindelin, J., Arganda-Carreras, I., Frise, E., Kaynig, V., Longair, M., Pietzsch, T., Preibisch, S., Rueden, C., Saalfeld, S., Schmid, B., et al. (2012). Fiji: an open-source platform for biological-image analysis. Nat. Methods *9*, 676–682. https://doi.org/10.1038/nmeth.2019.

Shi, Y., He, G., Lee, W.-C., McKenzie, J.A., Silva, M.J., and Long, F. (2017). Gli1 identifies osteogenic progenitors for bone formation and fracture repair. Nat. Commun. *8*, 2043. https://doi.org/10.1038/s41467-017-02171-2.

Sonoyama, W., Liu, Y., Yamaza, T., Tuan, R.S., Wang, S., Shi, S., and Huang, G.T.-J. (2008). Characterization of the apical papilla and its residing stem cells from human immature permanent teeth: a pilot study. J. Endod. *34*, 166–171. https://doi.org/10.1016/j.joen.2007.11.021.

Valenta, T., Degirmenci, B., Moor, A.E., Herr, P., Zimmerli, D., Moor, M.B., Hausmann, G., Cantù, C., Aguet, M., and Basler, K. (2016). Wnt ligands secreted by subepithelial mesenchymal cells are essential for the survival of intestinal stem cells and gut homeostasis. Cell Rep. *15*, 911–918. https://doi.org/10.1016/j.celrep.2016.03.088.

Vidal, V.P., Chaboissier, M.-C., Lutzkendorf, S., Cotsarelis, G., Mill, P., Hui, C.-C., Ortonne, N., Ortonne, J.-P., and Schedl, A. (2005). Sox9 is essential for outer root sheath differentiation and he formation of he hair stem cell comparmen. Current Biol. *9*, 1340–1351.

Wang, J., Jiang, Y., Xie, X., Zhang, S., Xu, C., Zhou, Y., and Feng, J.Q. (2022). The identification of critical time windows of postnatal root elongation in response to Wnt/β-catenin signaling. Oral Dis. *28*, 442–451. https://doi.org/10.1111/odi.13753.

Yang, J., Wang, S.K., Choi, M., Reid, B.M., Hu, Y., Lee, Y.L., Herzog, C.R., Kim-Berman, H., Lee, M., Benke, P.J., et al. (2015). Taurodontism, variations in tooth number and misshapen crowns in Wnt10a null mice and human kindreds. Mol. Genet. Genomic Med. *3*, 40–58.

Yang, S., Choi, H., Kim, T.H., Jeong, J.K., Liu, Y., Harada, H., and Cho, E.S. (2021). Cell dynamics in Hertwig's epithelial root sheath are regulated by β-catenin activity during tooth root development. J. Cell. Physiol. *236*, 5387–5398. https://doi.org/10.1002/jcp.30243.

Zeichner-David, M., Oishi, K., Su, Z., Zakartchenko, V., Chen, L.-S., Arzate, H., and Bringas, P. (2003). Role of Hertwig's epithelial root sheath cells in tooth root development. Dev. Dyn. *228*, 651–663. https://doi.org/10.1002/dvdy.10404.

Zhang, R., Yang, G., Wu, X., Xie, J., Yang, X., and Li, T. (2013). Disruption of wnt/β-catenin signaling in odontoblasts and cementoblasts arrests tooth root development in postnatal mouse teeth. Int. J. Biol. Sci. *9*, 228–236. https://doi.org/10.7150/ijbs.5476.

Zhao, H., Feng, J., Ho, T.-V., Grimes, W., Urata, M., and Chai, Y. (2015). The suture provides a niche for mesenchymal stem cells of craniofacial bones. Nat. Cell Biol. *17*, 386–396. https://doi.org/10.1038/ncb3139.

Zhao, J., Faure, L., Adameyko, I., and Sharpe, P.T. (2021). Stem cell contributions to cementoblast differentiation in healthy periodontal ligament and periodontitis. Stem Cells *39*, 92–102. https://doi.org/10.1002/stem.3288.

Zhong, Z., Zylstra-Diegel, C.R., Schumacher, C.A., Baker, J.J., Carpenter, A.C., Rao, S., Yao, W., Guan, M., Helms, J.A., Lane, N.E., et al. (2012). Wntless functions in mature osteoblasts to regulate bone mass. Proc. Natl. Acad. Sci. USA *109*, E2197–E2204.

Stem Cell Reports, Volume *18*

Supplemental Information

Wnt signaling from *Gli1*-expressing apical stem/progenitor cells is essential for the coordination of tooth root development

Rupali Lav, Jan Krivanek, Neal Anthwal, and Abigail S. Tucker

Supplemental Figures:

Figure S1:

***Axin2* lineage cells populate most mesenchymal cells of the incisor pulp and periodontium but not the epithelium**

(A) Schematic outlining the time course of tamoxifen (Tmx) administered to postnatal *Axin2*creERT2;R26mTmG mice for lineage tracing apical Wnt responding cells. (B) and magnified labial (B') and lingual (B'') cervical loops show sparce *Axin2* expressing cells (GFP) (24hrs post Tmx) found in the proximal incisor at P13. (C) and magnified labial (C') and lingual (C'') cervical loops show abundant *Axin2* lineage cells (GFP) populating the pulp and surrounding periodontium (PDL) but not the epithelial loops. n ≥ 3 tissue sections were examined from n= 5 mice. Scale bar in B,B' = 100μm, same scale in B' and C-C".

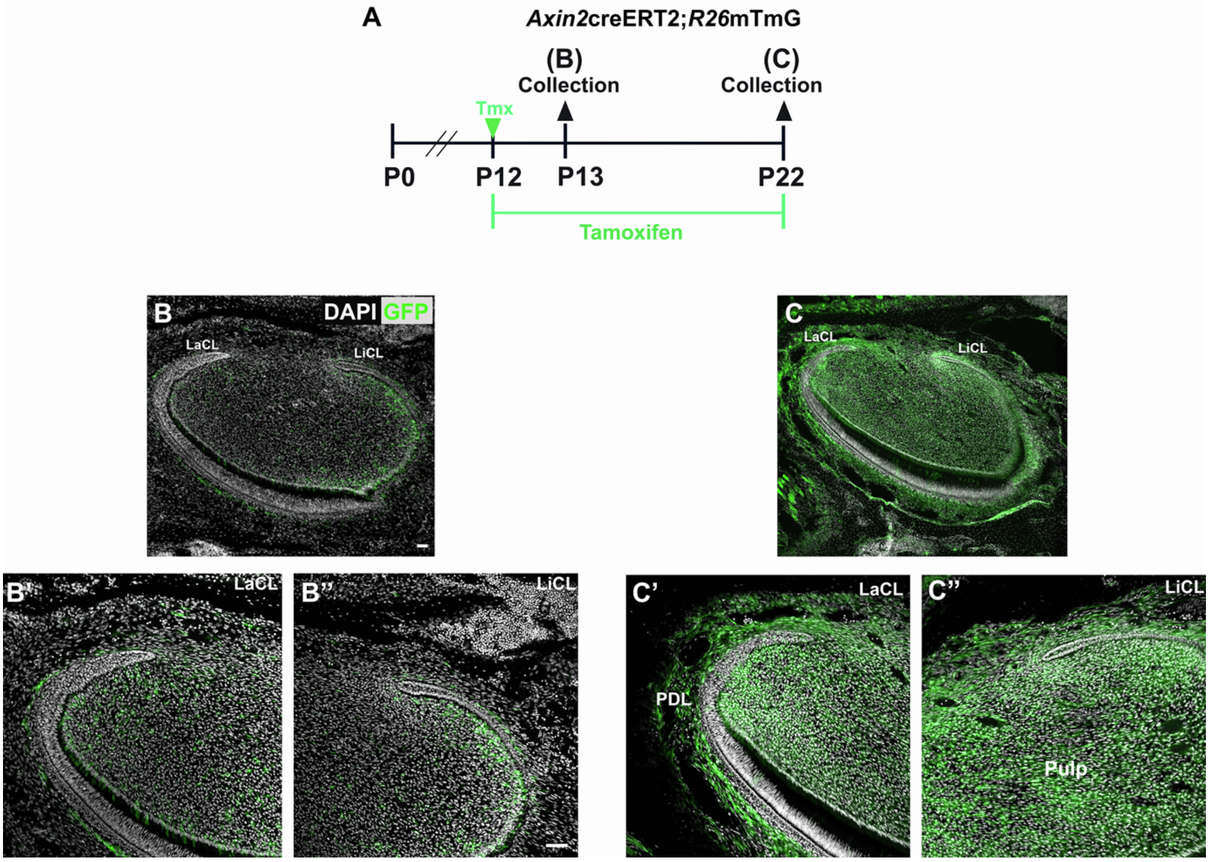

Figure S2:

Apical *Gli1*⁺ stem progenitor cells form all root lineage cells

(A) *Gli1*creERT2;R26Tomato reporter mice were used to labels stem/progenitor cells in the developing molar root. *Gli1* (detected through red fluorescent protein (RFP) expression- A') labels stem progenitor cells at the apex forming a proliferative (A''-Proliferative Cell Nuclear Antigen expression (PCNA)) progenitor cell population. Lineage traced *Gli1* progeny (RFP) (B and B'-magnified) form cells of all lineages in the root and surrounding periodontium (P-Pulp, Od-Odontoblasts, PDL-Periodontal Ligament, D-Dentin). n ≥ 3 tissue sections were examined from n= 3 mice. Scale bar in A", B and B'= 100µm, same scale in A-A'.

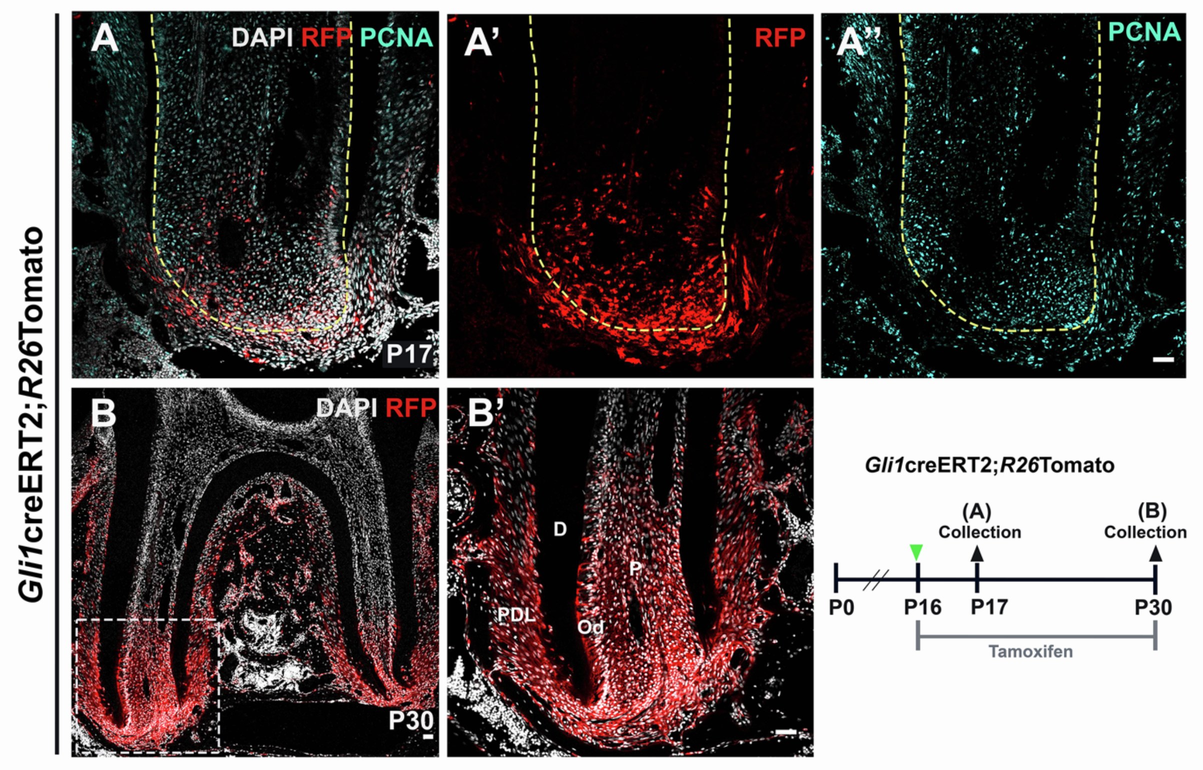

Figure S3:

Calvarial and molar defect in *Gli1*creERT2;*Wls*$^{fl/fl}$ mutant mice.

(A-C) RNAscope showing *Axin2* expression after loss of *Wntless* in the *Gli1* expressing population. (A) Apical region of molar (M1) P18 after tamoxifen at P13. (B) High power view of the apical region (box B in A) shows loss of *Axin2* expression (yellow dots). (C) High power view of periodontium (box C in A) shows high levels of *Axin2* expression (yellow dots). (D-I) microCT analysis of *Gli1creERT2;Wls*$^{fl/fl}$ mutant mice. Coronal (D,E) and sagittal (F-I). 3D microCT whole head reconstructions show that compared to littermate controls (D,F,H), *Gli1*creERT2;*Wls*$^{fl/fl}$ mutant mice exhibit rarefied bone especially adjacent to cranial sutures (E) and unerupted third molars (G), and truncated incisors (I). Asterix indicates defect in the lingual cervical loop. n= 5 mice. Scale bar in A-C = 50μm.

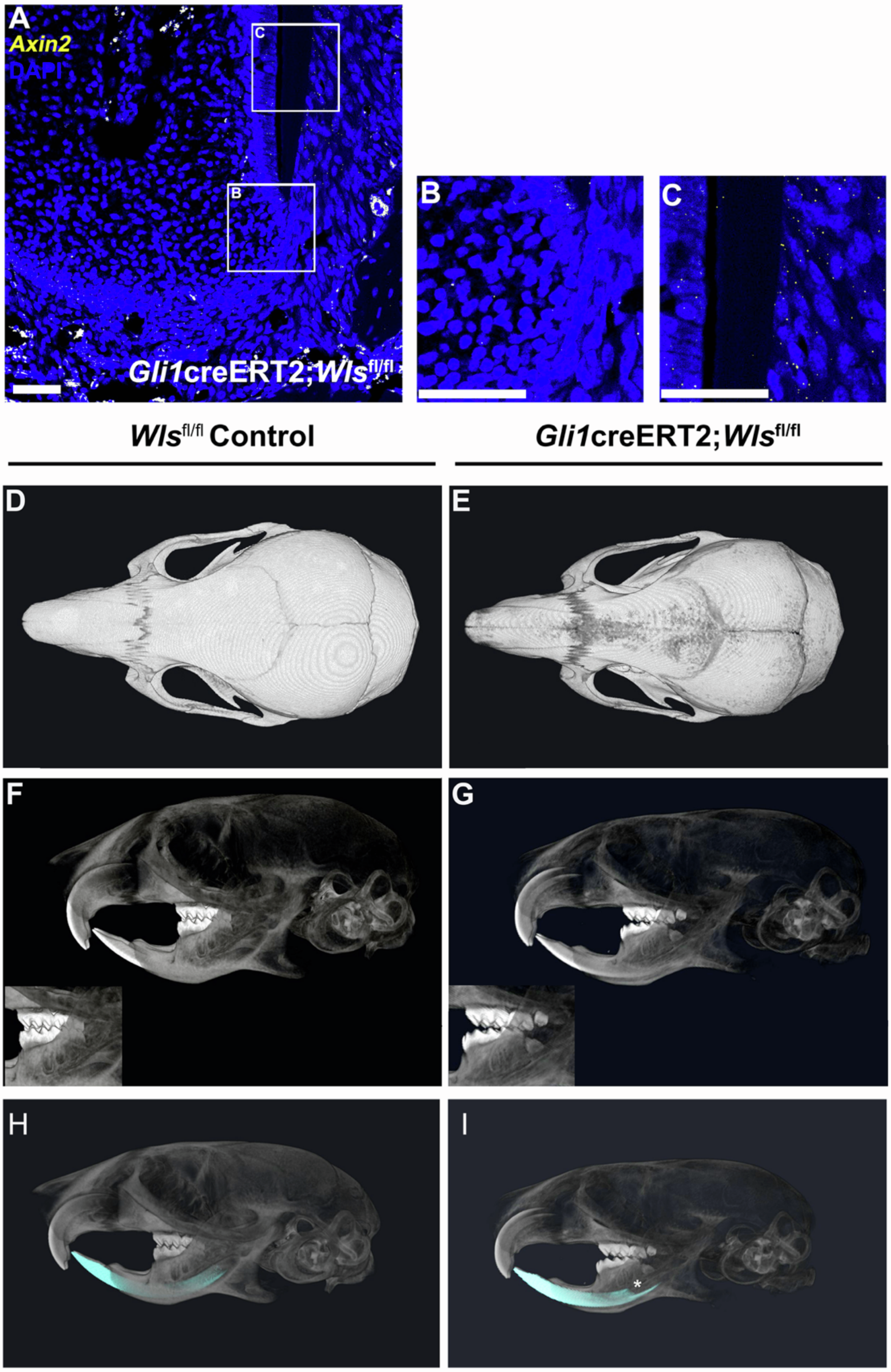

Figure S4:

Tooth root defects predate any evidence of bone defects at P18.

TRAP assay. Osteoclasts in red. (A,C,E,G) Wildtype littermate at P18, M1. (B,D,F,H) *Gli1creERT2;Wls$^{fl/fl}$* mutant mice at P18 after tamoxifen induction a P13, M1. Osteoclast activity appears normal at P18 after tamoxifen induction at P13. Scale bar in images = 50µm.

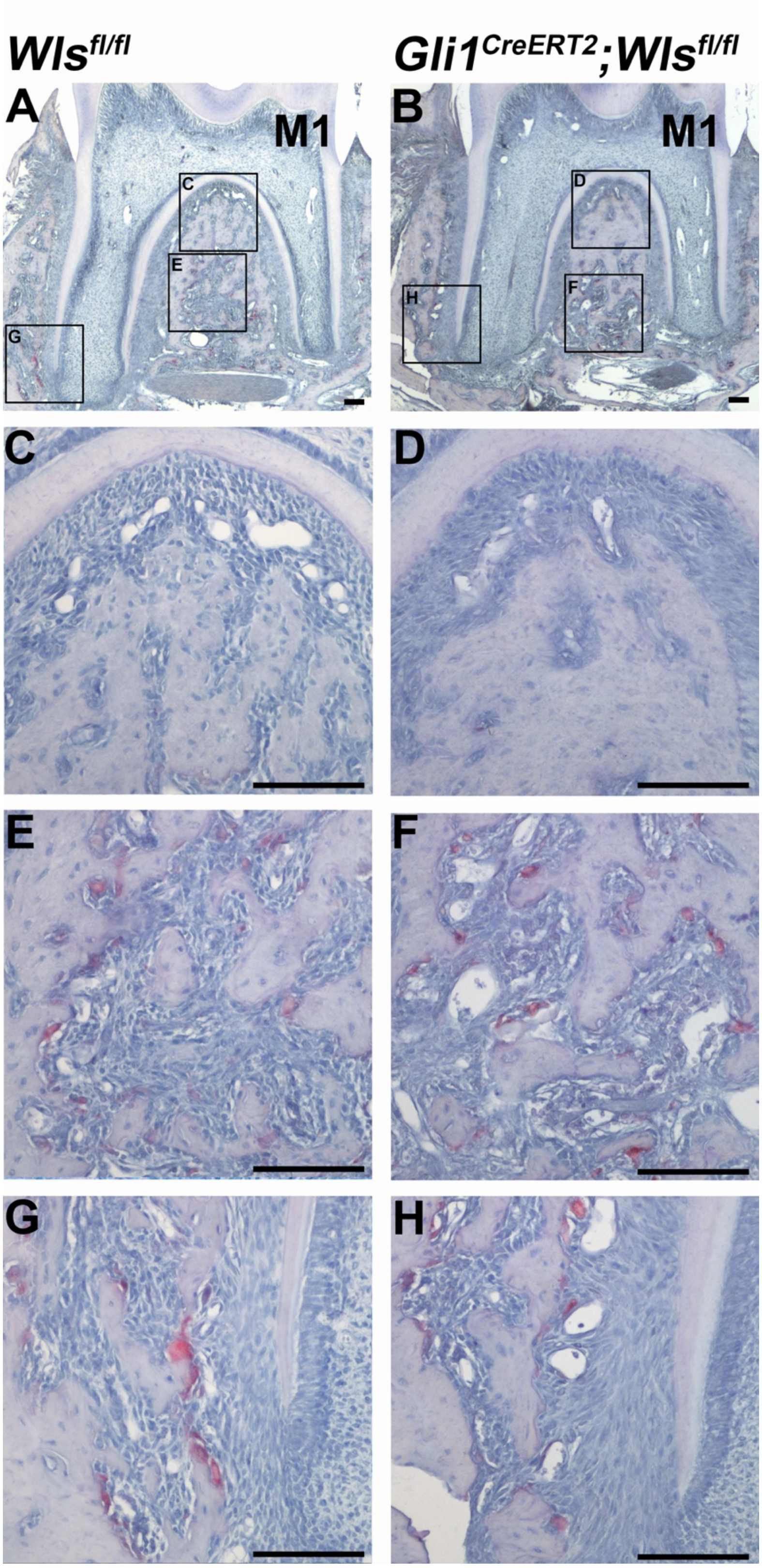

Figure S5:

Loss of Wnt ligands produced by *Gli1*$^+$ cells resulted in reduced cell proliferation

(A,B) P20. (C,D) P18. (A,A'- magnified) BrdU (5-Bromo-2'deoxyuridine) uptake (pink) by numerous proliferating apical root forming cells in non-Cre littermate controls (white arrowheads). (B, B'- magnified) Selective absence of cell proliferation in the apical root forming cells in mutants with *Wls* deletion in *Gli1*$^+$ cells (asterix). Unaffected proliferating cells are observed in the surrounding periodontium and under the HERS (Hertwig's Epithelial Root Sheath) (yellow arrowheads). (C, C') Proliferating cells (pink) in the control HERS (yellow arrowheads). Epithelial cells highlighted by pan-cytokeratin staining (green). (D,D') Lack of proliferating cells in the HERS in the mutant.
n ≥ 3 tissue sections were examined from n≥ 3 mice each from mutant and control samples. Scale bar in A, A' and C, C'= 100µm, same scale in B, B' and D,D'.

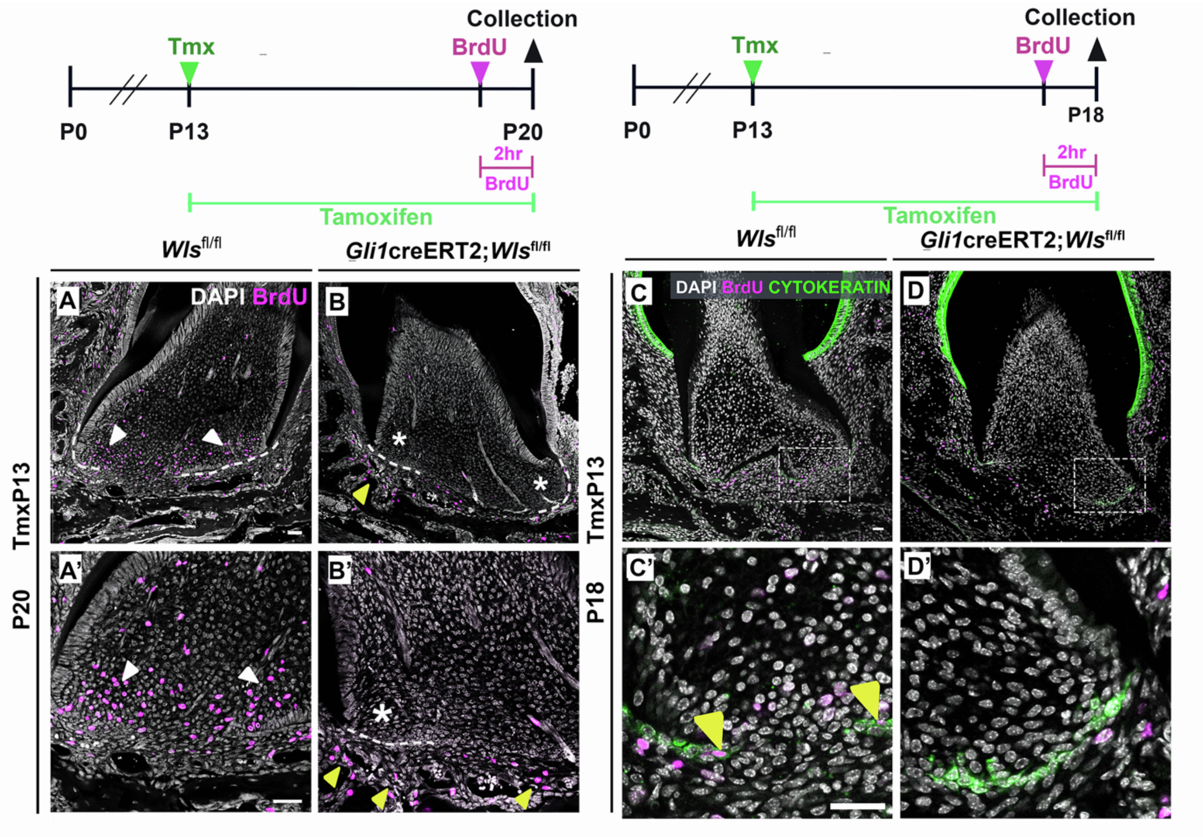

Figure S6:

Loss of Wnt ligands produced by *Gli1*⁺ cells resulted in loss of LAMININ expression and downregulation of Sox9 in the mutant HERS

LAMININ expression (pink) seen in the apical epithelium and mesenchyme of control developing roots (A, B-magnified) was lost in the mesenchyme of mutant molars (C,D-magnified). Low expression was seen in the apical HERS of mutant molars (D,D'). n ≥ 3 tissue sections were examined from n≥ 3 mice each from mutant and control samples

(E,G) Intact apical HERS cells of developing control molars labelled by E-CADHERIN (E-CAD) (green), with positive SOX9 cells (pink). Yellow arrowheads indicate SOX9 positive epithelial cells.

(F,H) Mutant HERS lose expression of SOX9 (pink) and E-CADHERIN (green) (blue arrowhead).

n ≥ 3 tissue sections were examined from n≥ 3 mice each from mutant and control samples. Scale bar in C,D,D',E,G= 100μm, same scale in A,B,B',F,H.

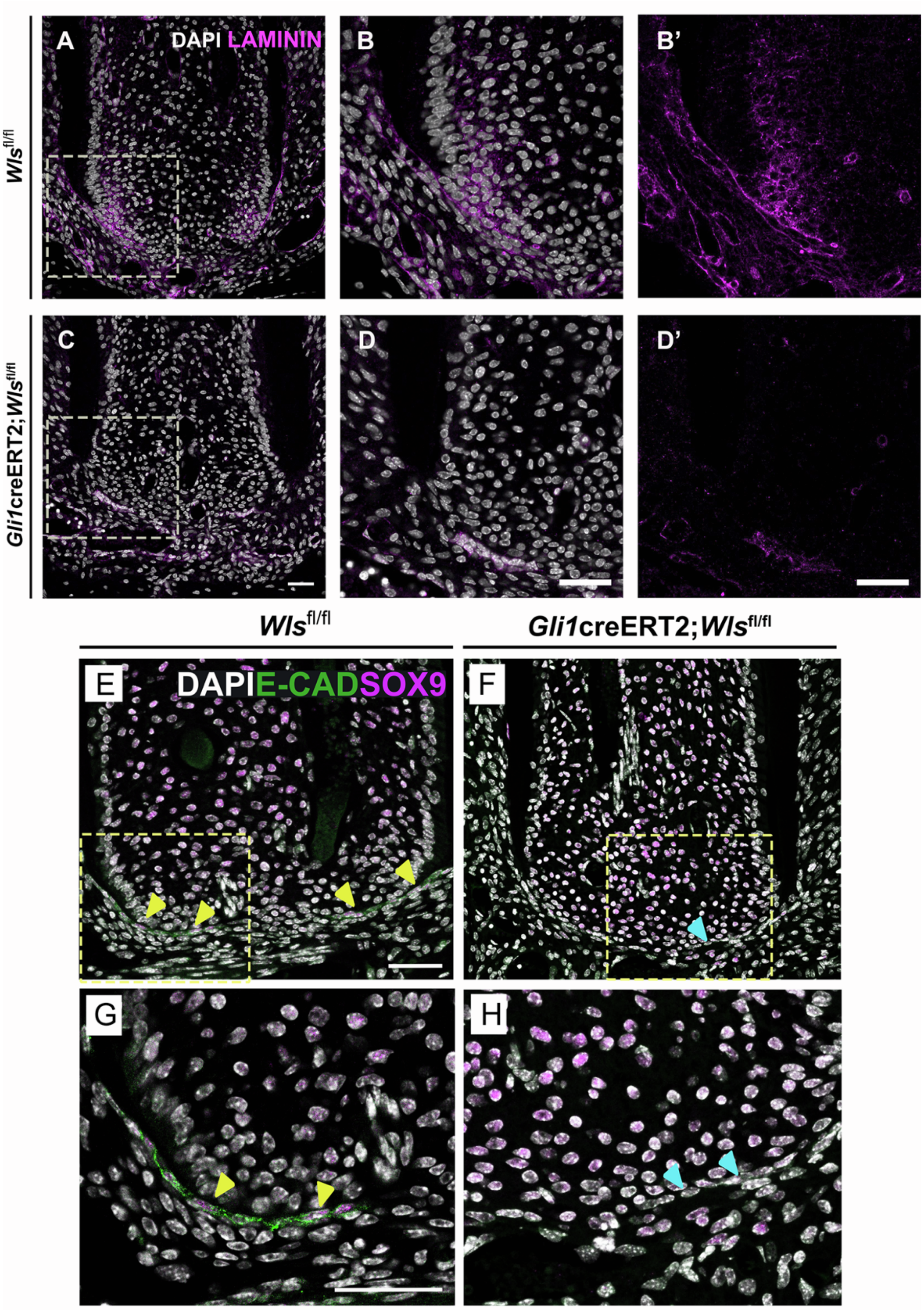

Figure S7:

***Sox9* deletion in *Gli1*$^{+}$ stem cells effects developing molars but not homeostatic incisors**

(A) Deletion of *Sox9* in *Gli1* expressing cells results in overall growth stunting accompanied by thinning of skin and hair loss. (B,C) Loss of SOX9 protein in *Gli1*creERT2;*Sox9*$^{fl/fl}$ mutant (C) compared to control littermates (B). As compared to non-Cre littermate controls (D,F) molar (haematoxylin and eosin stained histology sections) roots are severely affected in mutant teeth (E,G). Homeostatic incisors (microCT 3D reconstructions) however show no evident morphological difference between control (H,J) and mutant (I,K) samples. n=6 mice each with mutant (*Gli1*creERT2;*Sox9*$^{fl/fl}$) and control (non-Cre littermates). Scale bar in B = 50µm, same scale in C. Scale bar in D,F = 100µm, same scale in E,G.

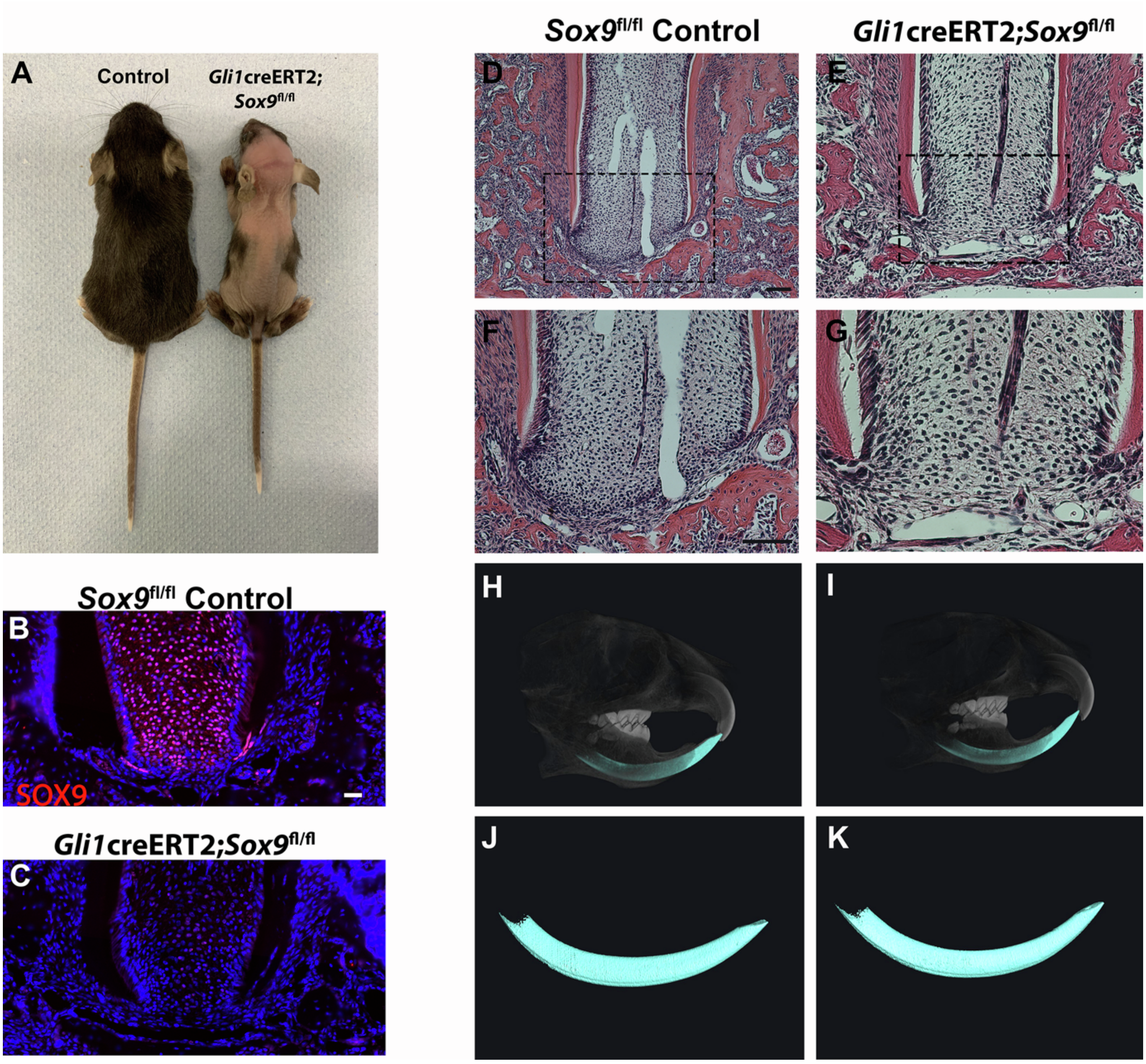

Supplemental Experimental methods:

Murine models:

All Cre mice were heterozygous for Cre, and loss of function experiments were performed in homozygous floxed models placed under temporally controlled Cre drivers. Gain of function experiments for constitutive overactivation of Wnt signaling were performed on heterozygous (*Ctnnb1*$^{lox(ex3)/+}$) mice.
For all inducible mouse lines, intraperitoneal injections of tamoxifen (Sigma T5648) dissolved in corn oil at 20 mg/ml with 10% ethanol were administered at indicated time points at a dose of 0.15 mg/g body weight. Additional BrdU (5-Bromo-2'deoxyuridine) (20mg/kg in normal saline) intraperitoneal injections were administered to *Gli1*creErt2;*Wls*$^{fl/fl}$ mice 2hrs prior to harvesting.

For all experiments, both female and male mice were non-preferentially used. Jaws were harvested at indicated time points post-induction and processed for further analysis.

Tissue processing and histological staining:

Mandibles with teeth were dissected and fixed overnight at 4°C in 4% paraformaldehyde (PFA), decalcified in 19% ethylenediaminetetraacetic acid (EDTA, pH 7.4), then dehydrated through an ascending series of graded ethanol, cleared with xylene, before wax infiltration with paraffin wax at 60°C. Paraffin wax-embedded samples were microtome sectioned at 8 µm thickness, then mounted on TruBond TM 380 adhesive slides. For histological examination, the slides were then stained with Hematoxylin and Eosin and/or picrosirius red trichrome stain using standard techniques. Stained sections were photographed using a Nikon eclipse 80i microscope.

X-gal staining and detection of β-galactosidase activity:

Postnatal day 16 (P16) murine *Axin2*LacZ mandibles were fixed in 0.2% glutaraldehyde overnight at 4°C, decalcified in 19% ethylenediaminetetraacetic acid (EDTA, pH 7.4) put through ascending graded sucrose series, embedded in OCT compound (Tissue-Tek) and sectioned with a cryostat at 10µm. For β-galactosidase (β-gal) activity detection, cryosections were washed in PBS, permeabilized in detergent rinse (1% Sodium Deoxycholate, 1M $MgCl_2$, 1% NP-40 in PBS, on ice for 10 min) and stained with X-gal staining solution [1M $MgCl_2$, 1 % sodium deoxycholate, 1% NP-40, 10 mM potassium ferricyanide, 10 mM potassium ferrocyanide, 1M Tris-HCL (pH 7.5) and 50 mg/ml X-gal (dissolved in Dimethyl formamide) in PBS] overnight at 37°C in a dark humidified chamber, followed by post-staining fixation in 4% PFA for 10 min at room temperature and counterstained with alcoholic Eosin histochemical stain. Stained sections were then flash dehydrated in ethanol, mounted in DPX and photographed using Nikon eclipse 80i microscope.

Immunofluorescence antibodies:

The following primary antibodies were used in this manuscript: 1/200 rabbit anti activated β-CATENIN (Cell Signaling, D13A1), 1/300 rabbit anti GFP (Invitrogen, A11122), 1/300 chicken anti GFP (Abcam ab13970), 1/300 rabbit anti PCNA (Abcam ab19166), 1/200 rat anti RFP (Chromotek, 5F8), 1/200 chicken anti GFP (Abcam, ab13970), 1/200 rat anti BrdU (Abcam, ab6326),1/200 rabbit anti pSMAD 1/5/8(Cell Signaling, D5B10), 1/300 rabbit anti CYOKERATIN (DAKO, Z0622), 1/200 mouse anti E-CADHERIN (Abcam, ab76055), 1/200 rabbit anti-LAMININ (Sigma Aldrich L9393), 1/200 rabbit anti SOX9 (Merck Millipore, AB5535), 1/200 goat anti-P-CADHERIN (R&D Systems, AF761;). Following TBS washes, sections were incubated with secondary antibody for 2 hours at room temperature. The following secondary antibodies were used at 1/300 concentration: Alexa568 conjugated Donkey anti-Rabbit (Invitrogen, A10042), Alexa 488 conjugated Donkey anti-Rabbit (Invitrogen, A21206), Alexa568 conjugated Donkey anti-Mouse (Invitrogen, A10037), Alexa594 conjugated Donkey anti-Rat (Invitrogen, A21209), Alexa488 conjugated Goat anti-Chicken (Invitrogen, A11039).
